# The gluconeogenesis enzyme PCK2 has a non-enzymatic role in proteostasis in endothelial cells

**DOI:** 10.1038/s42003-024-06186-6

**Published:** 2024-05-23

**Authors:** Pauline de Zeeuw, Lucas Treps, Melissa García-Caballero, Ulrike Harjes, Joanna Kalucka, Carla De Legher, Katleen Brepoels, Kristel Peeters, Stefan Vinckier, Joris Souffreau, Ann Bouché, Federico Taverna, Jonas Dehairs, Ali Talebi, Bart Ghesquière, Johan Swinnen, Luc Schoonjans, Guy Eelen, Mieke Dewerchin, Peter Carmeliet

**Affiliations:** 1 Laboratory of Angiogenesis and Vascular Metabolism, Department of Oncology, KU Leuven, Leuven, B-3000 Belgium; 2 Laboratory of Angiogenesis and Vascular Metabolism, Center for Cancer Biology, VIB, Leuven, B-3000 Belgium; 3 Laboratory of Lipid Metabolism and Cancer, Department of Oncology, KU Leuven, Leuven, B-3000 Belgium; 4 Metabolomics Core Facility, Department of Oncology, KU Leuven, Leuven, B-3000 Belgium; 5 Metabolomics Core Facility, Center for Cancer Biology, VIB, Leuven, B-3000 Belgium; 6 Center for Biotechnology, Khalifa University of Science and Technology, Abu Dhabi, United Arab Emirates; 7 Present Address: Droia Ventures, Zaventem, Belgium; 8 Present Address: CNRS, Nantes, France; 9 Present Address: Dept. Molecular Biology and Biochemistry, Fac. Science, University of Malaga, Malaga, Spain; 10 Present Address: Aarhus Institute of Advanced Studies (AIAS), Department of Biomedicine, Aarhus University, Aarhus, 8000 Denmark; 11 Present Address: Novartis Ireland, Dublin, Ireland; 12 Present Address: Metaptys NV/Droia Labs, Leuven, Belgium

**Keywords:** Angiogenesis, Biochemistry

## Abstract

Endothelial cells (ECs) are highly glycolytic, but whether they generate glycolytic intermediates via gluconeogenesis (GNG) in glucose-deprived conditions remains unknown. Here, we report that glucose-deprived ECs upregulate the GNG enzyme PCK2 and rely on a PCK2-dependent truncated GNG, whereby lactate and glutamine are used for the synthesis of lower glycolytic intermediates that enter the serine and glycerophospholipid biosynthesis pathways, which can play key roles in redox homeostasis and phospholipid synthesis, respectively. Unexpectedly, however, even in normal glucose conditions, and independent of its enzymatic activity, PCK2 silencing perturbs proteostasis, beyond its traditional GNG role. Indeed, PCK2-silenced ECs have an impaired unfolded protein response, leading to accumulation of misfolded proteins, which due to defective proteasomes and impaired autophagy, results in the accumulation of protein aggregates in lysosomes and EC demise. Ultimately, loss of PCK2 in ECs impaired vessel sprouting. This study identifies a role for PCK2 in proteostasis beyond GNG.

## Abstract (web summary)

This study reports a unexpected role in endothelial cells (ECs) for PCK2 in proteostasis, independent of its gluconeogenic activity. Perturbation of this role likely contributed to the angiogenic defects observed in ECs with reduced PCK2 levels.

## Introduction

Endothelial cells (ECs) form the inner lining of blood vessels. Emerging evidence reveals that EC metabolism controls vessel sprouting (angiogenesis) and that targeting EC metabolism can inhibit pathological angiogenesis^1–8^. ECs are highly glycolytic and use glycolytic intermediates for energy and biomass production and redox homeostasis^8^. In mature vessels, quiescent ECs are exposed to ample glucose in the plasma (5.5 mM). However, angiogenic ECs face a fluctuating nutrient supply and sprout into avascular areas, such as in tumor tissue in which glucose concentrations can be as low as 0.12 mM^9^. Whether and how ECs produce glycolytic intermediates in such glucose-deprived conditions, is unknown.

Gluconeogenesis (GNG), the process whereby non-carbohydrate carbon substrates (lactate, glycerol, amino acids (glutamine, alanine, serine, etc.)) are utilized to synthesize glucose that is then released to regulate blood glucose levels, typically occurs in the liver and, to a lesser extent, in kidneys^10^. In intestines and skeletal muscle, GNG produces glucose for internal use^10^, while in cancer cells, an abbreviated / truncated GNG (reverse glycolysis) pathway is used for the generation of glycolytic intermediates^11,12^. Next to reactions that are a reversal of glycolytic reactions, enzymes performing reactions specific to GNG involve pyruvate carboxylase (PC; converting pyruvate to oxaloacetate (OAA)), phosphoenol-pyruvate (PEP) carboxykinase (PEPCK, also termed PCK; converting OAA to PEP), fructose-1,6-bisphosphatase (FBP; converting fructose-1,6-bisphosphate (F1,6BP) to fructose-6-phosphate (F6P)), and glucose-6-phosphatase (G6Pase; converting glucose-6-phosphate (G6P) to glucose) (Supplementary Fig. 1a)^12,13^. A role for PCK isoform 2 (PCK2) in reverse glycolysis has been reported only in cancer cells^14–16^, where PCK2 fuels lipid and nucleotide synthesis in glucose-deprived conditions^11,12,14,15^.

Here, we explored the role of PCK2 in ECs, since, apart from data on effects on vascular smooth muscle cell proliferation^17^, PCK2’s function and relevance in non-transformed cells have not yet been studied, and ECs regularly utilize metabolic pathways / enzymes for different purposes than other (non-)malignant cell types^1,3–5,8^. Also, pilot experiments had revealed that silencing of PCK2 impaired vessel sprouting in normal glucose conditions (see below), thus raising the question whether PCK2 might have another distinct role in ECs, beyond its role in GNG (reverse glycolysis). We therefore studied the role of PCK2 in ECs in more detail, revealing a role for PCK2 in proteostasis, independent of its gluconeogenic activity. Pertubation of this role likely contributed to the angiogenic defects observed in vivo and in vitro in ECs with reduced PCK2 levels.

## Results

### Loss of endothelial PCK2 impairs vessel sprouting

**Fig. 1:**
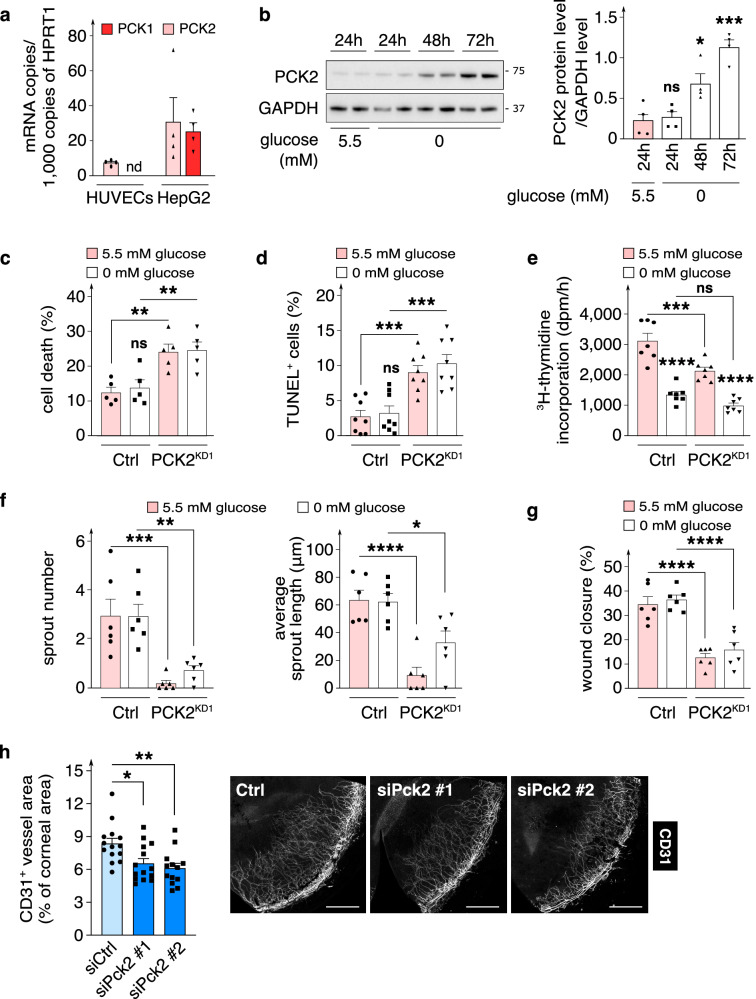
Role of endothelial PCK2 in vessel sprouting. **a** qRT-PCR analysis of *PCK1* and *PCK2* mRNA levels in ECs (HUVEC) (*n* = 4) and HepG2 cells (*n* = 4; used as positive control); nd, not detectable. **b** Representative immunoblot and densitometric quantification of PCK2 protein level in ECs in glucose-deprived (0 mM) *versus* control (24 h 5.5 mM glucose) conditions for the indicated time points (*n* = 4). GAPDH was used as a loading control. **c** Quantification of cell death (LDH release assay) in control and PCK2^KD1^ ECs in 5.5 *versus* 0 mM glucose (*n* = 5). **d** Quantification of TUNEL^+^ cells in control and PCK2^KD1^ ECs in 5.5 *versus* 0 mM glucose (*n* = 8). **e** ^3^H-thymidine incorporation into DNA in control and PCK2^KD1^ ECs in 5.5 *versus* 0 mM glucose (*n* = 7); dpm, disintegrations per minute. **f** Number of sprouts and average sprout length per spheroid in mitomycin C (MitoC)-treated control and PCK2^KD1^ ECs in 5.5 *versus* 0 mM glucose (*n* = 6). **g** Scratch wound closure (proxy of EC migration) in MitoC-treated control and PCK2^KD1^ ECs in 5.5 *versus* 0 mM glucose (*n* = 6). **h** Representative immunofluorescence images of CD31-stained neovessels (gray) in corneal flat-mounts from mice after surgical implantation in the eye of bFGF pellets containing a negative control or *Pck2*-targeting siRNA and corresponding quantification of CD31^+^ vessel area as percentage of total cornea area (*n* = 13–14). Scale bars are 300 µm. Data are mean ± s.e.m. Statistics: ANOVA (**c**–**g**), two-tailed *t*-test with Welch’s correction (**b**, **h**); *P < 0.05; **P < 0.01; ***P < 0.001; ****P < 0.0001; ns not significant.

In normal (5.5 mM) glucose conditions, human umbilical vein ECs (HUVECs; used in most experiments and as such referred to as ECs from hereon) expressed the mitochondrial isoform PCK2, while the cytosolic isoform PCK1 was undetectable (Fig. 1a; Supplementary Fig. 1b, c). PCK2 protein was detected in the mitochondrial fraction, as assessed by co-fractionation with the mitochondrial-specific voltage-dependent anion channel (VDAC1) protein (Supplementary Fig. 1b). Moreover, immunostaining revealed that PCK2 co-localized with TOMM20, a mitochondrial-specific protein (Supplementary Fig. 1c), further confirming that PCK2 is localized in mitochondria in ECs. In contrast, both PCK isoforms were readily detected in a hepatocyte cell line (Fig. 1a). Glucose-deprivation (0 mM) time-dependently elevated PCK2 protein expression (Fig. 1b), consistent with reports in other non-EC types^14^. A similar increase in PCK2 expression upon glucose-deprivation was observed in human umbilical arterial ECs (HUAECs) and microvascular ECs (HMVECs) (Supplemenatry Fig. 1d).

We studied the functional role of PCK2 in vessel sprouting in vitro in both normal and glucose-deprived conditions by lentiviral transduction of a control (Ctrl) *versus PCK2*-specific shRNA expressing vector (PCK2^KD1^ or PCK2^KD2^, referring to independent, non-overlapping shRNAs). ECs were exposed to glucose deprivation during 48 h, i.e. sufficiently long to upregulate PCK2 expression, without however compromising EC viability (Fig. 1c, d; Supplementary Fig. 1e). Silencing of *PCK2* with PCK2^KD1^ lowered *PCK2* mRNA and protein levels by ~85% (Supplemenatry Fig. 1f, g). Glucose deprivation impaired EC proliferation to such an extent that PCK2^KD1^ did not further reduce EC proliferation (likely because proliferation was already maximally reduced by glucose deprivation) (Fig. 1e; Supplementary Fig. 1h). The readout of the vessel sprouting assay (using EC spheroids) as well as the wound closure assay (scratch wounds in EC monolayers) depends not only on EC migration but also on EC proliferation^18^. To score the effects of PCK2 silencing on EC migration separately, and since the effect of PCK2 silencing on EC proliferation could not be evaluated upon glucose deprivation (see above), we first pretreated ECs with mitomycin C (MitoC; an anti-mitotic agent inhibiting DNA synthesis) to eliminate the effect of EC proliferation. Glucose deprivation itself did not affect migration or vessel sprouting of MitoC-treated ECs, while PCK2^KD1^ impaired angiogenic behavior of glucose-deprived ECs (Fig. 1f, g). PCK2^KD1^ also reduced the viability of glucose-deprived ECs (Fig. 1c,d; Supplementary Fig. 1e). Qualitatively similar results were obtained in arterial ECs (PCK2^KD1^ HUAECs) (Supplementary Fig. 2a–d) and in ECs transduced with the second shRNA sequence targeting PCK2 (PCK2^KD2^) (lowering *PCK2* mRNA and protein levels by up to 90%; Supplementary Fig. 1g; 2e-i). We typically used PCK2-silenced ECs cultured in 10 mM glutamine (present in basal EC growth medium) but obtained qualitatively comparable results when using medium containing 2 mM glutamine (typically used in in vitro cancer studies^14,19–21^) or 0.6 mM (levels measured in human plasma^22^) (Supplementary Fig. 2j–m). Throughout this study (see below), key findings in PCK2^KD1^ ECs were additionally verified and confirmed in PCK2^KD1^ HUAECs, PCK2^KD2^ ECs and PCK2^KD1^ ECs cultured in lower glutamine concentrations (see Supplementary Figures).

To assess whether endothelial PCK2 affects angiogenesis in vivo, we used an established pre-clinical model for pathological corneal neovascularization involving surgical implantation of slow-release pellets containing basic fibroblast growth factor (bFGF) in the cornea of the mouse to induce growth of blood vessels into the otherwise avascular cornea^23^. Inclusion of either of two different, non-overlapping *Pck2*-targeting siRNAs (with 88-94% silencing efficiency; Supplementary Fig. 2n) in the bFGF pellet significantly reduced the formation of CD31^+^ blood vessels in the cornea (Fig. 1h) to a comparable extent as observed upon blocking of other metabolic genes^4,5,24^.

### PCK2 silencing affects EC morphology and membrane integrity

**Fig. 2:**
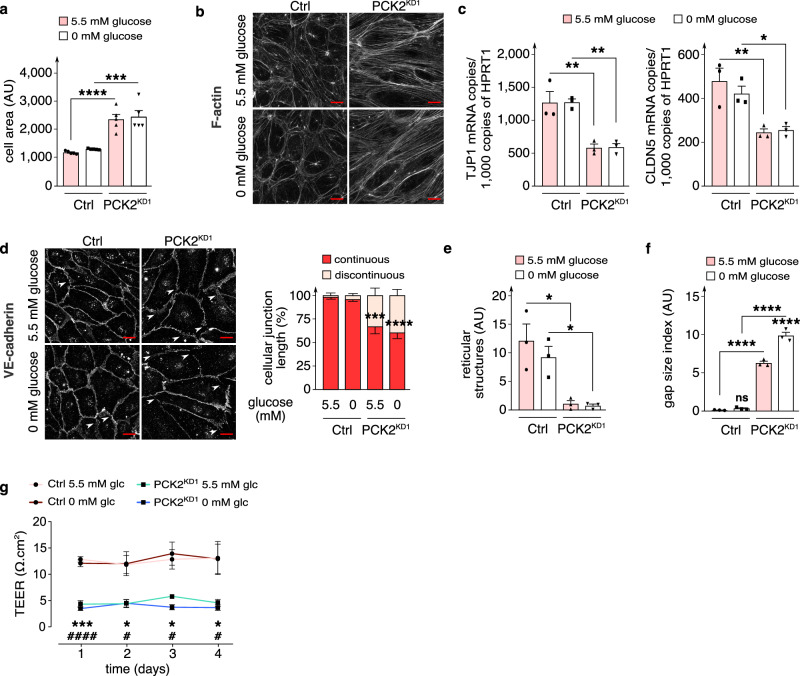
Effect of PCK2 silencing on EC morphology and membrane integrity. **a** Quantification of cell area in control and PCK2^KD1^ ECs in 5.5 *versus* 0 mM glucose (*n* = 5). **b** Representative immunofluorescence images of F-actin (phalloidin staining; white) in control and PCK2^KD1^ ECs in 5.5 *versus* 0 mM glucose (*n* = 5). **c** qRT-PCR analysis of tight junction protein 1 (*TJP1*) and claudin-5 (*CLDN5*) mRNA levels in control and PCK2^KD1^ ECs in 5.5 *versus* 0 mM glucose (*n* = 3). **d** Representative immunofluorescence images of VE-cadherin staining and quantification of VE-cadherin^+^ (dis)continuous junction length (graph right to the images) in control and PCK2^KD1^ ECs in 5.5 *versus* 0 mM glucose (*n* = 3). White arrowheads show VE-cadherin^+^ discontinuous junctions. **e**, **f** Quantification of reticular structure area (**e**) and gap size index (**f**) on VE-cadherin immunostained control and PCK2^KD1^ ECs in 5.5 *versus* 0 mM glucose (*n* = 3). **g** Trans-endothelial electrical resistance (TEER) measurements in proliferation-blocked (MitoC-treated) confluent control and PCK2^KD1^ EC monolayers in 5.5 *versus* 0 mM glucose (glc) (*n* = 5). Asterisks and hashtags in (**g**) denote statistically significant differences between KD and control respectively at 5.5 mM and at 0 mM glucose. Data are mean ± s.e.m. Statistics: ANOVA (**a**, **c**–**f**) two-tailed *t*-test with Welch’s correction (**g**); * or ^#^*P* < 0.05; ** or ^##^*P* < 0.01; *** or ^###^*P* < 0.001; **** or ^####^*P* < 0.0001; ns not significant. Scale bars in (**b**, **d**) are 10 µm; AU, arbitrary units.

**Fig. 3:**
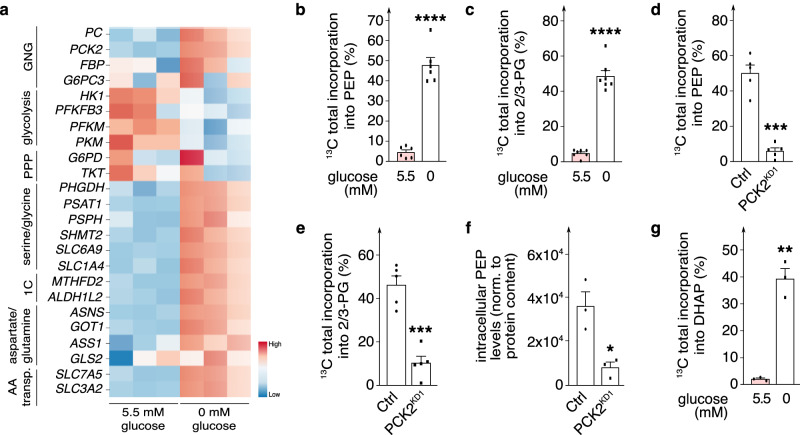
PCK2 controls channeling of glutamine- and lactate-derived carbons into lower glycolytic intermediates in glucose-deprived ECs. **a** Heatmap of transcript levels of metabolic genes involved in gluconeogenesis (GNG), glycolysis, pentose phosphate pathway (PPP), serine and glycine biosynthesis pathway, 1-carbon (1 C) metabolism, aspartate and glutamine metabolism and amino acid (AA) transporters (transp.) from bulk RNA sequencing of ECs in 5.5 *versus* 0 mM glucose (*n* = 3). Color scale: red, high expression; blue, low expression. **b**, **c** Incorporation of [U^13^C]-glutamine and [U^13^C]-lactate carbon into total intracellular phosphoenolpyruvate (PEP) pool (**b**; *n* = 6) and 2/3-phosphoglycerate (2/3-PG) pool (**c**; *n* = 7) in ECs in 5.5 *versus* 0 mM glucose. **d**, **e** Incorporation of [U^13^C]-glutamine and [U^13^C]-lactate carbon into total intracellular PEP pool (**d**) and 2/3-PG pool (**e**) in control and PCK2^KD1^ glucose-deprived ECs (*n* = 5). **f** Intracellular levels of PEP, normalized (norm.) to protein content (expressed in AUC/µg protein), in control and PCK2^KD1^ glucose-deprived ECs (*n* = 3). AUC, area under the curve. **g** Incorporation of [U^13^C]-glutamine and [U^13^C]-lactate carbon into total intracellular dihydroxyacetone phosphate (DHAP) pool in ECs in 5.5 *versus* 0 mM glucose (*n* = 3). Data are mean ± s.e.m. Statistics: two-tailed *t*-test with Welch’s correction (**b**–**g**); *P < 0.05; **P < 0.01; ***P < 0.001; ****P < 0.0001.

Additional in vitro phenotyping revealed that, irrespective of the extracellular glucose concentrations, PCK2^KD1^ enlarged EC size (Fig. 2a; Supplemenatry Fig. 3a, b) (without inducing senescence), perturbed the F-actin cytoskeleton and evoked actin stress fibers (Fig. 2b), and disrupted cellular membrane integrity and barrier function as evidenced by: (i) decreased expression of tight junction protein 1 and claudin-5 (involved in barrier formation^25^) (Fig. 2c; Supplementary Fig. 3c, d); (ii) decreased VE-cadherin^+^ continuous junction length and increased discontinuous junction length (Fig. 2d), reduced area of reticular junctions with a honeycomb staining pattern (contributing to barrier function^26^) and increased intercellular gap area (Fig. 2e, f); and (iii) reduced trans-endothelial electrical resistance (TEER) (Fig. 2g). Such effects of PCK2^KD1^ on the cell size, morphology and plasma membrane integrity have not been previously reported in malignant cells^14,15^.

Surprisingly, however, PCK2 silencing similarly impaired angiogenic behavior, enlarged EC size and impaired vascular barrier integrity, when ECs were cultured in 5.5 mM glucose (Figs. 1c–g, 2a–g), raising the question whether PCK2 might have an activity, beyond GNG / reverse glycolysis (which is mainly / only active in glucose-deprived conditions). In an attempt to explain the activity spectrum of PCK2, we first investigated its metabolic role in glucose-deprived conditions and thereafter characterized its role in normal glucose conditions.

### PCK2 controls channeling of glutamine- and lactate-derived carbons into lower glycolytic intermediates in glucose-deprived ECs

We explored to which extent GNG / reverse glycolysis is operational in glucose-deprived ECs. RNA-sequencing and qRT-PCR analyses revealed that glucose deprivation upregulated the expression of *PC* and *FBP*, while lowering the transcript levels of glycolytic and non-oxidative pentose phosphate pathway (PPP) genes (Fig. 3a; Supplementary Fig. 1a and 4a). *G6PC3* (G6Pase isoform) transcript levels were not affected by glucose concentrations (Fig. 3a; Supplementary Fig. 1a). Interestingly, glucose deprivation elevated the expression of genes involved in serine / glycine biosynthesis, one carbon metabolism, and amino acid transport (Fig. 3a) (see below). In agreement, mass-spectrometry measurements revealed that intracellular pools of glycolytic and PPP metabolites were reduced upon glucose deprivation, while intracellular levels of multiple amino acids were elevated (Supplementary Fig. 4b). Interestingly, most of these amino acids are gluconeogenic substrates^27^.

We performed stable isotope tracer experiments to assess whether glucose-deprived ECs channel carbons from GNG substrates (glutamine, lactate) into glycolytic intermediates to feed into glycolytic side pathways. Of note, glutamine (single letter code Q) is the most abundant amino acid in the human body and cancer cells rely heavily on tumor microenvironment-derived glutamine for their survival in glucose-deprived conditions^19,28^. Lactate (single letter code L) was used at a concentration within the range detected in experimental tumors^29^. When ECs were exposed to the combination of [U^13^C]-glutamine (10 mM) and [U^13^C]-lactate (5 mM) (^13^C-Q + L), up to 5% of the total intracellular PEP pool contained ^13^C label in normal glucose-exposed ECs, increasing to 48% in glucose-deprived ECs (Fig. 3b). Similar incorporations were observed in the upstream glycolytic intermediate 2/3-phospoglycerate (2/3-PG) (Fig. 3c). As expected, glucose deprivation lowered the intracellular pools of these and other glycolytic intermediates (Supplementary Fig. 4b). Hence, in the absence of extracellular glucose, a larger fraction of glycolytic intermediates could be derived from alternative carbon sources, such as lactate and glutamine, than in the presence of glucose.

PCK2^KD1^ in glucose-deprived ECs reduced ^13^C-Q + L-derived label incorporation into cellular PEP and 2/3-PG pools (Fig. 3d, e), confirming that glucose-deprived ECs rely on PCK2 to produce PEP from OAA. These data were validated by treatment of glucose-deprived ECs with 3-mercaptopicolinic acid (3-MPA), a PCK pharmacological inhibitor^30^ (Supplementary Fig. 4c, d). A similar reduction was obtained in lower glutamine concentrations (Supplementary Fig. 4e, f). Of note, incorporation of ^13^C label from ^13^C-Q + L into TCA cycle metabolites (precursors of GNG / reverse glycolysis) was not affected by PCK2 inhibition (PCK2^KD1^; 3-MPA) (Supplementary Fig. 4g, h), suggesting that the reduced ^13^C-Q + L-derived label incorporation into intracellular PEP and 2/3-PG pools upon PCK2 blockade was specifically due to inhibition of the enzymatic activity of PCK2 rather than to a lower supply of precursors. As expected, PCK2^KD1^ lowered the intracellular pool of PEP in glucose-deprived ECs (Fig. 3f).

Exploration of ^13^C-Q + L-derived carbon enrichment in metabolites upstream of 2/3-PG in glucose-deprived ECs revealed ^13^C label incorporation in dihydroxyacetone phosphate (DHAP) (Fig. 3g), but negligibly (not above background) in upstream glycolytic intermediate hexoses (F6P, G6P) or ribose-5-phosphate of the (oxidative) pentose phosphate pathway (PPP) to which G6P connects (Supplementary Fig. 4i, j). Overall, these data illustrate that ECs express GNG enzymes, at higher levels upon glucose deprivation, and that PCK2 regulates in ECs a truncated GNG (reverse glycolysis) pathway, which does not serve the purpose of glucose production and secretion (as in liver and kidney^10^) or nucleotide biosynthesis via the PPP (as in cancer cells^31,32^). We thus explored alternative fates of the truncated GNG pathway.

### PCK2-derived glycolytic intermediates shuttle into the serine / glycine biosynthesis pathway

**Fig. 4:**
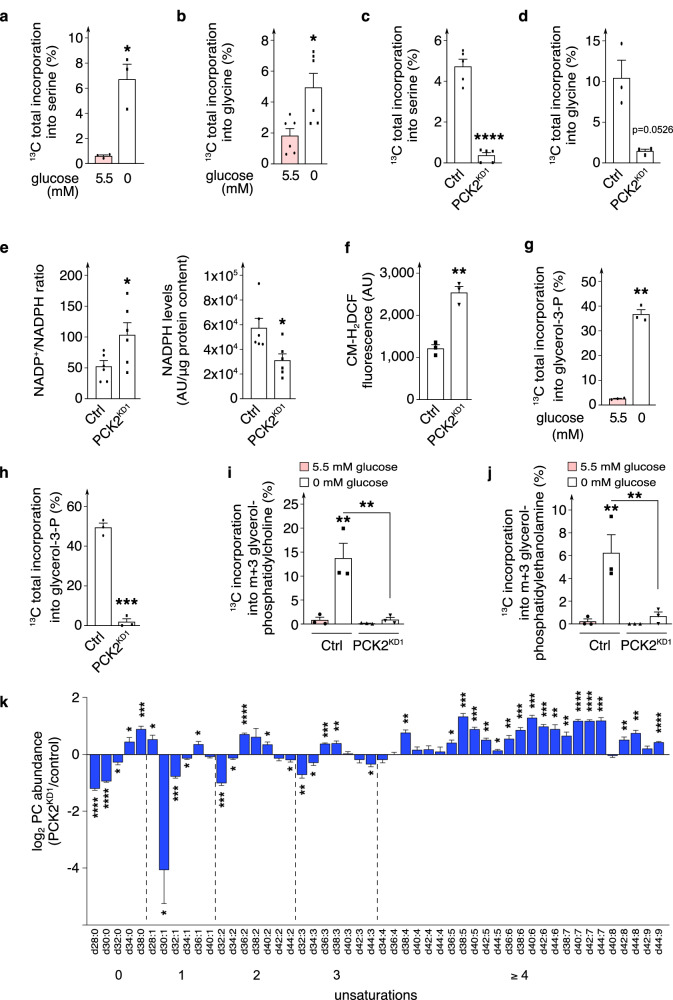
PCK2-derived glycolytic intermediates shuttle into serine/glycine biosynthesis and glyceroneogenesis. **a**, **b** Incorporation of [U^13^C]-glutamine and [U^13^C]-lactate carbon into total intracellular serine pool (**a**; *n* = 3) and glycine pool (**b**; *n* = 6) in ECs in 5.5 *versus* 0 mM glucose. **c**, **d** Incorporation of [U^13^C]-glutamine and [U^13^C]-lactate carbon into total intracellular serine pool (**c**; *n* = 5) and glycine pool (**d**; *n* = 3) in control and PCK2^KD1^ glucose-deprived ECs. **e** Intracellular NADPH levels and NADP^+^/NADPH ratio in control and PCK2^KD1^ glucose-deprived ECs (*n* = 6). **f** Quantification of cellular ROS levels (measured as median CM-H_2_DCF fluorescence levels) in control and PCK2^KD1^ glucose-deprived ECs (*n* = 3). **g** Incorporation of [U^13^C]-glutamine and [U^13^C]-lactate carbon into total intracellular glycerol-3-phosphate pool in ECs in 5.5 *versus* 0 mM glucose (*n* = 3). **h** Incorporation of [U^13^C]-glutamine and [U^13^C]-lactate carbon into total intracellular glycerol-3-phosphate pool in control and PCK2^KD1^ glucose-deprived ECs (*n* = 3). **i**, **j** Incorporation of [U^13^C]-glutamine and [U^13^C]-lactate carbon into fractional m + 3 intracellular glycerol-phosphatidylcholine pool (**i**) and glycerol-phosphatidylethanolamine pool (**j**) in control and PCK2^KD1^ ECs in 5.5 *versus* 0 mM glucose (*n* = 3). **k** Phospholipidomic profile analysis showing the log_2_ abundance of saturated, mono-, di- and poly-unsaturated glycerol-phosphatidylcholine (PC) phospholipid species in PCK2^KD1^ glucose-deprived ECs (*n* = 4–5 HUVEC donors) relative to their respective wild type control glucose-deprived ECs. The numbers under the axes indicate the carbon length of the fatty acid within each group of unsaturation degree (zero to ≥ 4). Data are mean ± s.e.m. Statistics: two-tailed *t*-test with Welch’s correction (**a**–**h**); ANOVA (**i**, **j**); one-sample *t* and Wilcoxon test (**k**) *P < 0.05; **P < 0.01; ***P < 0.001; ****P < 0.0001. AU arbitrary units.

Glucose deprivation upregulated the expression of enzymes in the serine / glycine biosynthesis pathway (phosphoglycerate dehydrogenase [*PHGDH*], phosphoserine amino-transferase 1 [*PSAT1*], serine hydroxymethyltransferase isoform 2 [*SHMT2*], which converts serine into glycine) (Fig. 3a; Supplementary Fig. 5a). Since 3-PG is a precursor of serine synthesis, we hypothesized that PCK2 in glucose-deprived ECs regulated serine and glycine synthesis from substrates other than glucose. Indeed, in glucose-deprived ECs, ^13^C-Q + L-derived ^13^C incorporation into serine and glycine was increased (Fig. 4a, b), which was largely ablated by PCK2^KD1^ or 3-MPA treatment (Fig. 4c, d; Supplementary Fig. 5b, c). Notably, PCK2 silencing also lowered the expression of *PHGDH*, *PSAT1* and *SHMT2* (Supplementary Fig. 5d–f). In cancer cells, glucose deprivation upregulates serine biosynthesis and one carbon metabolism to stimulate nucleotide synthesis for cellular proliferation^15^. However, unlike cancer cells, which continue to proliferate upon glucose deprivation^15^, glucose-deprived ECs proliferate less, and thus do not need to employ the serine biosynthesis pathway and one carbon metabolism for nucleotide synthesis. Since serine / glycine biosynthesis serves additional functions (production of glutathione and NADPH^33,34^), we explored if PCK2 silencing affected redox homeostasis. Indeed, PCK2 silencing lowered total NADPH levels, elevated the NADP^+^/NAPDH ratio (Fig. 4e) and increased intracellular reactive oxygen species (ROS) (Fig. 4e, f; Supplementary Fig. 5g). Thus, glucose-deprived ECs may, at least in part, rely on a PCK2-dependent truncated GNG for redox homeostasis. Increased ROS levels are known to disrupt EC junctions and to impair EC barrier integrity and vessel sprouting^2,35^.

### PCK2-derived glycolytic intermediates shuttle into the glyceroneogenesis pathway

We also noticed that glucose-deprived ECs showed ^13^C-Q + L-derived tracer enrichment in glycerol-3-phosphate (Fig. 4g), which was reduced upon PCK2 blockade (Fig. 4h; Supplementary Fig. 5h). PCK2^KD1^ also reduced intracellular levels of glycerol-3-phosphate (Supplementary Fig. 5i), which is the 3-carbon backbone for fatty acid esterification to form diacyl- and triacyl-glycerols (the latter are also known as triglycerides for fat storage), and glycerol-phospholipids, the main structural component of biological membranes^36^. As synthesis of triglycerides compared to glycerol-phospholipids is low in ECs^36^ and is unlikely to serve fat storage in lipid droplets in glucose deprivation, as evidenced by the absence of lipid droplets in ECs (Supplementary Fig. 5j), we hypothesized that ^13^C-Q + L-derived carbons could be traced into glycerol-phospholipids rather than triglycerides. Indeed, in glucose-deprived ECs, we detected m + 3 label incorporation of up to 14% into phosphatidylcholine (PC) and 6% into phosphatidylethanolamine (PE), two major glycerol-phospholipid classes in ECs^36,37^ (Fig. 4i, j). This incorporation was not detected in ECs cultured in normal glucose concentrations (likely because glucose is normally the source of these lipids), nor in glucose-deprived PCK2^KD1^ ECs (Fig. 4i, j). Phospholipidomics revealed that cellular levels of saturated and mono-unsaturated PC phospholipid species were decreased in PCK2^KD1^ ECs, while levels of poly-unsaturated PC phospholipid species were increased (Fig. 4k). The shift towards polyunsaturated glycerol-phospholipids may reflect a compensatory increased uptake of extracellular polyunsaturated fatty acids, which can be modified intracellularly, upon a decrease of lipogenesis^38^. Gene expression of glycerol-3-phosphate acyltransferase (*GPAM*; involved in the first committed and rate-limiting step of glycerol-phospholipid synthesis by converting glycerol-3-phosphate into lyso-phosphatidic acid) was also decreased in PCK2-silenced ECs (Supplementary Fig. 5k–m). Levels of sphingomyelin (another phospholipid that contains sphingosine instead of glycerol-3-phosphate as backbone) did not differ between control and PCK2^KD1^ ECs (Supplementary Fig. 5n), strengthening the role of PCK2 in glyceroneogenesis for the synthesis of glycerol-phospholipids in ECs upon glucose deprivation. Overall, glucose-deprived ECs used non-carbohydrate carbon substrates for the synthesis of glycerol-phospholipids. Impaired lipid biosynthesis in glucose-deprived PCK2-silenced ECs may thus also contribute to their defective EC barrier integrity^36,39^. Even though a qualitatively similar role for PCK2 has been documented in cancer cells^14^, an effect of PCK2 silencing on cellular barrier integrity has not been reported yet (see also Discussion).

### PCK2-silenced ECs have decelerated autolysosomal clearance of protein aggregates

**Fig. 5:**
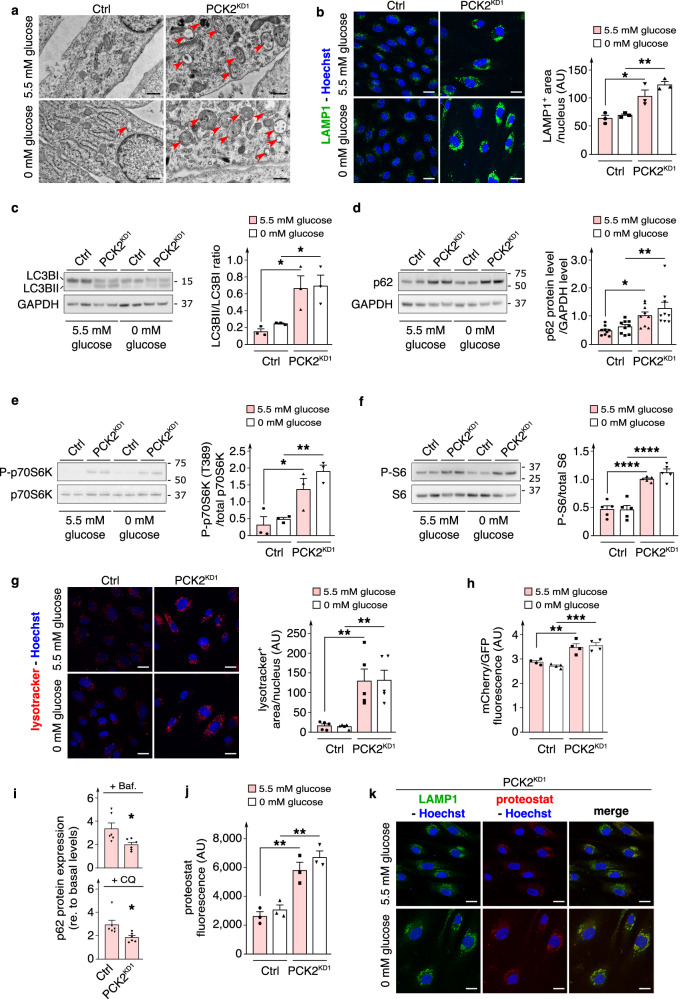
PCK2-silenced ECs have decelerated autolysosomal clearance of protein aggregates. **a** Representative transmission electron microscopy (TEM) images of control and PCK2^KD1^ ECs in 5.5 *versus* 0 mM glucose (*n* = 3). Red arrowheads show (auto)lysosomal structures. **b** Representative immunofluorescence images of LAMP1 (green; lysosomal marker) staining and quantification of LAMP1^+^ staining area per cell nucleus in control and PCK2^KD1^ ECs in 5.5 *versus* 0 mM glucose (*n* = 3). Nuclei are counterstained with Hoechst (blue). **c** Representative immunoblot of LC3B protein level and densitometric quantification of LC3BII (lipidated)/LC3BI (non-lipidated) ratio in control and PCK2^KD1^ ECs in 5.5 *versus* 0 mM glucose (*n* = 3). GAPDH was used as a loading control. **d** Representative immunoblot and densitometric quantification of p62 protein level in control and PCK2^KD1^ ECs in 5.5 *versus* 0 mM glucose (*n* = 9). GAPDH was used as a loading control. **e** Representative immunoblot and densitometric quantification of phosphorylated (P)-p70S6K at threonine (T) 389/total p70S6K protein ratio in control and PCK2^KD1^ ECs in 5.5 *versus* 0 mM glucose (*n* = 3). **f** Representative immunoblot and densitometric quantification of phosphorylated (P)-S6/total S6 protein ratio in control or PCK2^KD1^ ECs in 5.5 *versus* 0 mM glucose (*n* = 5). **g** Representative images of lysotracker (red) and Hoechst (blue) staining and quantification of lysotracker^+^ staining area per cell nucleus in control and PCK2^KD1^ ECs in 5.5 *versus* 0 mM glucose (*n* = 5). **h** Quantification of relative autolysosomal degradation levels (measured as the ratio of median mCherry/GFP fluorescence levels) in control and PCK2^KD1^ ECs transduced with mCherry(acid-insensitive)-GFP(acid-sensitive)-LC3 lentiviral vector (see Methods) in 5.5 *versus* 0 mM glucose (*n* = 4). **i** Quantification of p62 protein level in control and PCK2^KD1^ ECs in normal glucose conditions, upon 2 h treatment with bafilomycin A (Baf.; 50 nM) or chloroquine (CQ; 50 µM) (relative to untreated control or PCK2^KD1^ ECs) (*n* = 6). **j** Quantification of protein aggregate levels (measured as median proteostat fluorescence levels) in control and PCK2^KD1^ ECs in 5.5 *versus* 0 mM glucose (*n* = 3). **k** Representative immunofluorescence images of LAMP1 (green), proteostat (red) and Hoechst (blue) staining in PCK2^KD1^ ECs in 5.5 *versus* 0 mM glucose (*n* = 3). Data are mean ± s.e.m. Statistics: ANOVA (**b**–**h**, **j**), two-tailed *t*-test with Welch’s correction (**i**); *P < 0.05; **P < 0.01; ***P < 0.001; ****P < 0.0001. Scale bars are 500 nm in (**a**) and 10 µm in (**b**, **g**, **k**); AU arbitrary units.

Oddly, two other observations in PCK2-silenced ECs could not be attributed to the traditional metabolic role of PCK2 in GNG / reverse glycolysis, and suggested another distinct role of PCK2 in EC biology: (i) first, the finding that PCK2 silencing induced similar phenotypes in normal and zero extracellular glucose concentrations; and (ii) the finding that PCK2 silencing enlarged (doubled) EC size. To study the underlying mechanisms of the cell size enlargement, we characterized cellular morphology in greater detail. Transmission electron microscopy revealed extensive accumulation of lysosomes and autophagosomes in PCK2^KD1^ ECs, regardless of extracellular glucose concentrations (Fig. 5a). Stainings for the lysosomal membrane protein LAMP-1 confirmed that PCK2^KD1^ ECs contained more lysosomes than control ECs, in either glucose condition (Fig. 5b). Lysosomes fuse with autophagosomes to form autolysosomes that, in turn, degrade autophagosome content. Interestingly, levels of the lipidated autophagosome marker LC3B (LC3BII; causing an increased LC3BII/LC3BI ratio) and autophagic substrate p62 (Fig. 5c, d; Supplementary Fig. 6a–d) were increased in PCK2^KD^ ECs, suggesting impaired lysosomal degradation and autophagic flux. Accordingly, PCK2^KD1^ ECs upregulated the activity of mTORC1 (an inhibitor of autophagic flux^40^) as measured by the increased phosphorylation of its downstream target p70S6K and its substrate S6 (Fig. 5e, f). Treatment of PCK2^KD1^ ECs with 50 nM rapamycin, an autophagy inducer via mTORC1 inhibition, resulted in a partial reduction of accumulated p62 protein expression (Supplementary Fig. 6e).

Live-imaging of ECs labeled with LysoTracker, a fluorescent probe that stains acidic organelles such as lysosomes, confirmed the increased number of lysosomes in PCK2^KD1^ ECs, and revealed that these lysosomes had the required acidity for proteolytic activity (Fig. 5g). Overexpression of LC3 fused to both GFP and mCherry^41,42^ revealed that PCK2^KD1^ ECs contained numerous lysosomes that had efficiently fused with autophagosomes to form autolysosomes but had not degraded their contents (Fig. 5h; Supplementary Fig. 6f). In response to a 2-h treatment with inhibitors of lysosomal acidification (50 nM bafilomycin A; 50 µM chloroquine), PCK2^KD1^ ECs showed a smaller additional increase in p62 accumulation than control ECs, validating a slower degradation of autophagosome content by PCK2^KD1^ endothelial lysosomes (Fig. 5i). Thus, although autophago-lysosomal fusion remained operational and lysosomes of PCK2^KD1^ ECs were acidified, they degraded their contents relatively slowly at steady state, possibly leading to accumulation of debris.

Aggregated proteins can be concentrated at perinuclear sites of aggregate deposition, referred to as aggresomes^43^. Aggresomes provide a cytoprotective function by sequestering toxic denatured / misfolded aggregated proteins and facilitate their ultimate elimination by autolysosomal degradation^43^. Staining denatured / misfolded aggregated proteins with Proteostat revealed that PCK2^KD1^ ECs accumulated protein aggregates, as quantified by flow cytometry (Fig. 5j). Co-staining of Proteostat with anti-LAMP-1 revealed that the majority of these protein aggregates were contained within perinuclear lysosomes (Fig. 5k), consistent with the impaired autophagic flux and slow autolysosomal degradation in PCK2^KD^ ECs.

### Silencing of PCK2 affects proteostasis

**Fig. 6:**
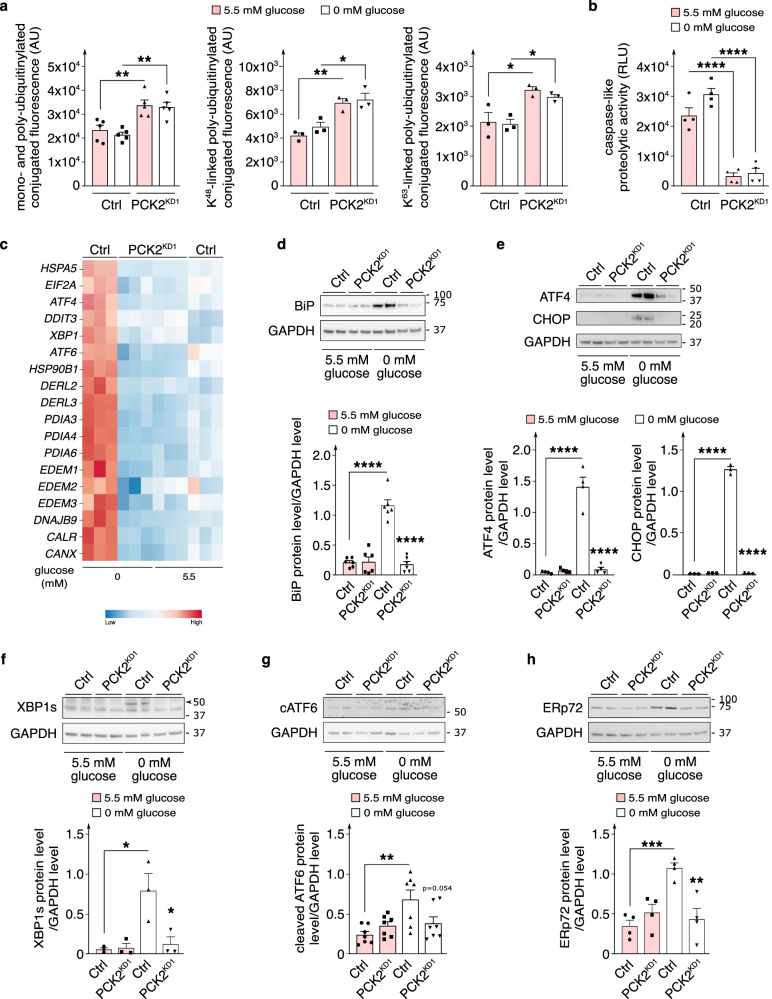
PCK2 silencing affects proteostasis. **a** Quantification of conjugated ubiquitin levels (measured as median fluorescence levels of mono- and poly-ubiquitinylated conjugates (*n* = 5), specific K48-linked poly-ubiquitinylated conjugates (*n* = 3) or specific K63-linked poly-ubiquitinylated conjugates (*n* = 3)) in control and PCK2^KD1^ ECs in 5.5 *versus* 0 mM glucose. AU, arbitrary units. **b** Quantification of proteasome activity (measured as luminescence signal emitted upon the degradation of aminoluciferin-tagged peptide substrate Z-nLPnLD-aminoluciferin upon caspase-like proteolytic activity) in control and PCK2^KD1^ ECs in 5.5 *versus* 0 mM glucose (*n* = 4); RLU relative luminescence units. **c** Heatmap of transcript levels of molecular chaperones (BiP (HSPA5), eIF2α (EIF2A), ATF4, CHOP (DDIT3), XBP1, ATF6, heat shock protein 90 (HSP90B1), Derlin (DERL2/3), PDI (PDIA3/4/6), EDEM1/2/3, DNAJB9, calreticulin (CALR) and calnexin (CANX)) involved in the unfolded protein response (UPR) assessed by bulk RNA sequencing of control and PCK2^KD1^ ECs in 5.5 *versus* 0 mM glucose (*n* = 3). Color scale: red, high expression; blue, low expression. **d**–**h** Representative immunoblot and densitometric quantification of BiP (**d**; *n* = 6), ATF4 (**e**; *n* = 4), CHOP (**e**; *n* = 3), spliced XBP1 (XBP1s; see black arrowhead) (**f**; *n* = 3), cleaved ATF6 (cATF6) (**g**; *n* = 7) and ERp72 (**h**; *n* = 4) protein levels in control and PCK2^KD1^ ECs in 5.5 *versus* 0 mM glucose. GAPDH was used as a loading control. Data are mean ± s.e.m. Statistics: ANOVA (**a**, **b–h**); *P < 0.05; **P < 0.01; *** *P* < 0.001; ****P < 0.0001.

Aggresomes form when the ubiquitin-proteasome machinery is overloaded with aggregation-prone denatured / misfolded proteins. Conjugated ubiquitin levels (mono- and poly-ubiquitinylated conjugates; specific K48-linked poly-ubiquitinylated conjugates; specific K63-linked poly-ubiquitinylated conjugates) were increased in PCK2^KD1^ ECs (Fig. 6a), indicating higher levels of misfolded proteins that received a poly-ubiquitin chain to target them for proteasomal or autolysosomal degradation. However, PCK2^KD1^ ECs showed reduced proteasome activity, as indicated by the decreased levels of luminescence signal emitted upon degradation of the aminoluciferin-tagged peptide substrate Z-nLPnLD by one of the main proteolytic activities of the S20 proteasome core (caspase-like proteolytic activity) (Fig. 6b). Thus, PCK2-silenced ECs accumulated poly-ubiquitin-tagged misfolded proteins, which due to dysfunctional proteasomal degradation, resulted in a build-up of aggregation-prone denatured / misfolded proteins, consistent with the increase in protein aggregates. Denatured / misfolded protein accumulation perturbs protein homeostasis (proteostasis), essential for normal cellular function.

In addition to proteasomal and autolysosomal degradation, molecular chaperones are used to maintain proteostasis by facilitating the recognition, refolding or degradation of misfolded proteins – their levels are typically upregulated by endoplasmic reticulum (ER) stress and the unfolded protein response (UPR). To explore whether PCK2 also regulates this process, we therefore deprived PCK2^KD1^ ECs of glucose (known to trigger ER stress and to induce UPR^44^; compare ctrl ECs in normal glucose to ctrl ECs in zero glucose in Fig. 6c) and performed exploratory unbiased RNA-sequencing profiling. Indeed, glucose-deprived PCK2^KD1^ ECs reduced the expression of molecular chaperones (including heat shock proteins (HSPA5/BiP; HSP90B1), involved in degradation of ER protein (DERL2/3), protein disulfide isomerases (PDIA3/4/6), calreticulin (CALR) and calnexin (CANX)) that facilitate the recognition, refolding or degradation of misfolded proteins to levels detected in PCK2^KD1^ ECs in normal glucose (Fig. 6c), indicating that sufficient PCK2 levels are necessary to trigger an ER stress response and induce UPR. Moreover, protein levels of ATF4, spliced XBP1 (XBP1s) and cleaved ATF6 (cATF6), key ER-stress related UPR transcription factors^45^, including upstream UPR-regulator protein BiP and downstream UPR-mediator proteins CHOP and ERp72, were all decreased in glucose-deprived PCK2^KD1^ and PCK2^KD2^ ECs (Fig. 6d–h; Supplementary Fig. 7a–f). Similarly, PCK2^KD1^ ECs in normal glucose conditions treated with ER stressor thapsigargin (2 µM) for 6 h revealed reduced protein expression of BiP, ATF4, XBP1s, cATF6 and ERp72 (Supplementary Fig. 8a–e). Thus, in conditions that induce cellular and ER stress and subsequent accumulation of misfolded proteins, PCK2-silenced ECs have an impaired UPR and consequently a perturbed protein homeostasis.

## Discussion

Our study revealed (i) additional metabolic roles of PCK2 in glucose-deprived ECs as compared to other cell types; and (ii) a role of PCK2 in the ER stress response and UPR, beyond its metabolic activity, irrespective of glucose availability.

Our findings reveal that in glucose-deprived ECs, PCK2 regulates an abbreviated GNG / reverse glycolysis pathway, utilizing non-carbohydrate carbon substrates to fuel (i) the serine / glycine biosynthesis pathway, which is involved in redox control; and (ii) the glyceroneogenesis pathway, which provides the backbone for glycerol-phospholipid synthesis, essential for endothelial membrane biogenesis^36^. The increased oxidative stress and impaired glycerol-phospholipid synthesis in glucose-deprived PCK2-silenced ECs may contribute to their impaired angiogenic behavior and vascular barrier integrity. A role of PCK2 in glucose-deprived conditions in securing cellular barrier integrity has not been reported before. While we and others previously identified an essential role of fatty acid oxidation in securing vascular barrier maintenance in normal glucose conditions^2,46^, we identify here a metabolic mechanism (PCK2-regulated GNG / reverse glycolysis) to secure vascular barrier integrity in glucose-deprived conditions in vitro. Whether and to which extent this mechanism secures barrier integrity in vivo, requires further study.

Another striking and entirely unexpected outcome of our study was the finding of an additional and previously unrecognized role of PCK2 in cellular proteostasis, beyond its traditional metabolic role in GNG / reverse glycolysis. Indeed, PCK2^KD^ impaired the angiogenic behavior of ECs even in normal glucose conditions. Mechanistically, irrespective of extracellular glucose concentrations, PCK2 silencing in ECs perturbed cellular proteostasis causing aggravated (yet unresolved) ER stress and subsequent accumulation of toxic protein aggregates in lysosomes. These aggregates can cause extensive cellular damage, ultimately resulting in cellular demise, in itself sufficient to explain the impaired angiogenic behavior and vascular barrier integrity defect of PCK2^KD^ ECs in normal glucose conditions. To our knowledge, an essential role for PCK2 in proteostasis, in sustaining an ER stress response and supporting the UPR, in ECs (or even in any other cell type) has not yet been described. Remarkably, unlike PCK2 silencing, blocking PCK2’s catalytic activity with 3-MPA does not impair UPR or perturb protein homeostasis in glucose-deprived ECs (no reduction in BiP and ATF protein levels, no accumulation of LAMP1 or protein aggregates) (Supplementary Fig. 9a–c) and did not reduce EC proliferation or migration or increase EC death (Supplementary Fig. 9d–f). These findings suggest an uncoupling of the above phenomena from PCK2’s catalytic activity but require additional research efforts for formal confirmation/negation (e.g. through construction and use of fully catalytic dead PCK2 mutants in ECs).

An outstanding question is how mitochondrial PCK2 is involved in the (mainly) ER-confined UPR. Recent reports have uncovered a signaling axis between mitochondrial perturbation and the ER stress response, such as ATF4 induction^47,48^. Of interest, ATF4 transcriptionally upregulates PCK2 in cancer cells^21^; oppositely, we now show that in ECs upon ER stress (e.g. thapsigargin treatment or glucose-deprivation), PCK2 silencing prevents ATF4 induction. Given that ATF4 transcriptionally regulates autophagy^49^, this lack of ATF4 induction in PCK2-silenced ECs (even in baseline conditions; without thapsigargin treatment or glucose-deprivation) may contribute to the observed decelerated autolysosomal clearance / impaired autophagic flux. Another possible mechanistic link may relate to the close physical contacts between mitochondria and a specialized ER domain, known as the mitochondria-associated membrane (MAM). A number of ER protein folding chaperones are present in MAMs and alterations in MAM integrity and signaling have been linked to the disruption of proteostasis^50^. Interestingly, three independent proteomic studies documented PCK2 in the MAM fraction^51–53^. More evidence for a non-enzymatic role by PCK isoforms, beyond GNG, is the recently reported protein kinase activity of PCK1 resulting in the phosphorylation of INSIG1/2 proteins and subsequent promotion of cancer cell growth^54^.

ECs and multiple cancer cells express PCK2, at elevated levels in glucose-deprived conditions, but otherwise, both cell types exhibit several differences. First, although GNG enzymes were previously assumed to be absent from cancer cells that did not arise in gluconeogenic organs (liver, kidney), several studies demonstrated their expression in diverse cancers as mediators of abbreviated forms of GNG^11,12,55^. A role of GNG in ECs was not known. Both glucose-deprived ECs and cancer cell types exhibit an abbreviated GNG, but cancer cells utilize reverse glycolysis for nucleotide synthesis by diverting glycolytic intermediates into serine biosynthesis pathway and PPP (relying on the rate-controlling GNG enzyme FBP)^11,12^, while ECs shunt glycolytic intermediates into the serine biosynthesis pathway, but carbons from non-carbohydrate substrates were not detected in PPP-generated riboses in ECs. This may relate to the findings that many transformed cancer cells are genetically reprogrammed to continuously proliferate even in nutrient-deprived conditions^11^, while non-transformed ECs adapt their metabolism in response to the availability of nutrients. Second, PCK2 was recently reported to restrict abundance of and ^13^C-glutamine incorporation into tricarboxylic acid cycle (TCA) intermediates, curbing mitochondrial respiration in cancer cells and preserving reduced glutathione levels and redox potential^56^. In contrast, PCK2-silencing did not increase ^13^C-Q + L incorporation in TCA intermediates in ECs; our results rather suggest that PCK2-dependent GNG may support normal redox balance via shuttling generated glycolytic intermediates into the serine / glycine biosynthesis pathway and downstream glutathione production. Third, whereas the glyceroneogenesis pathway also supported de novo glycerol-phospholipid synthesis in both ECs (this study) and cancer cells, the majority of non-carbohydrate-derived carbon incorporation was found in phosphatidylcholine in ECs rather than in phosphatidylethanolamine in cancer cells^14^. Fourth, while PCK2 silencing impaired survival in ECs, cancer cells display more context-dependence on PCK2 levels: *PCK2* overexpression reduced tumorigenesis of liver cancer cells^57^ and proliferation of renal carcinoma cells^58^, whereas *PCK2* silencing reduced colony formation of human lung cancer cells^56^ and proliferation of breast cancer cell lines^21,59^, but PCK2 inhibition enhanced survival of tumor initiating cells of melanoma^60^, thus illustrating the contextual role of PCK2 in different cell types. Fifth, further supporting the different role of GNG enzymes in cancer cells *versus* ECs, both cancer cells and ECs utilize GNG enzymes for functions beyond their traditional role in synthesizing glycolytic intermediates, but rely on different GNG enzymes. Indeed, cancer cells use FBP to suppress cancer cell growth partly by non-enzymatic mechanisms^11,13,61^, while in ECs, PCK2 promotes growth, vessel barrier integrity and sprouting, partly through a role beyond its traditional role in GNG.

Overall, we identified in this study additional metabolic roles of PCK2 in abbreviated GNG in glucose-deprived ECs, different from PCK2’s role in cancer cells, and provide evidence for a previously unrecognized role of PCK2 in proteostasis, beyond its traditional activity in GNG, irrespective of glucose concentrations. Both mechanisms likely contributed to the angiogenic defects in vitro and in vivo in ECs with reduced PCK2 levels. The results also suggests that endothelial PCK2 might be considered a therapeutic target for anti-angiogenic medicine.

## Methods

### Cell lines and culture

#### Primary human macrovascular umbilical endothelial cells

Human umbilical vein endothelial cells (HUVECs) and human umbilical artery endothelial cells (HUAECs) were freshly isolated from umbilical cords obtained from healthy donors (with approval from the Medical Ethical Committee KU Leuven / UZ Leuven (protocol S57123) and informed consent obtained from all subjects) as previously described^3^. The endothelial cells (ECs) were initially cultured and maintained in M199 medium (1 mg/mL D-glucose) (Thermo Fisher Scientific) supplemented with 20% fetal bovine serum (FBS) (Thermo Fisher Scientific), 2 mM L-glutamine (Thermo Fisher Scientific), 30 mg/L Endothelial Cell Growth Supplement (ECGS) (PromoCell), 10 units/mL heparin (PromoCell), 100 IU/mL penicillin and 100 µg/mL streptomycin (Thermo Fisher Scientific). For experimental procedures, ECs were cultured in either basal endothelial cell growth medium 2 (EGM-2) (PromoCell) supplemented with SupplementMix (PromoCell) or in specifically requested customized EGM-2 medium (PromoCell) (without glucose, pyruvate, glutamine, lactate, serine and alanine) supplemented with EGM supplement pack (PromoCell), except for FCS-10 which was replaced by 2% dialyzed FBS (Thermo Fisher Scientific). Further, 1 mM sodium pyruvate (Thermo Fisher Scientific), 10 mM L-glutamine, 5 mM sodium L-lactate (Sigma-Aldrich), 0.3 mM serine (Sigma-Aldrich) and 0.03 mM alanine (Sigma-Aldrich) were added. All experiments including glucose-deprived ECs had ECs cultured in 0 mM *versus* 5.5 mM glucose-containing EGM-2 for at least 48 h, unless otherwise stated. To obtain 5.5 mM glucose EGM-2 medium, 5.5 mM D-glucose (Sigma-Aldrich) was added to glucose-free (0 mM) EGM-2 medium. Depending on the experimental procedure, other metabolites or chemical compounds added to the medium include 1 µg/mL mitomycin C (MitoC; 16 h treatment) (Sigma-Aldrich), 100 µM 3-mercaptopicolinic acid (3-MPA; 48 h treatment) (Santa Cruz Biotechnology), 50 nM bafilomycin A (2 h treatment) (Santa Cruz Biotechnology), 50 µM chloroquine (2 h treatment) (Thermo Fisher Scientific), 50 nM rapamycin (24 h treatment) (InVivoGen), 2 µM thapsigargin (6-h treatment) (Sigma-Aldrich). When chemical compounds were dissolved in dimethyl sulfoxide (DMSO) (Sigma-Aldrich), an equal volume of DMSO was added to the medium of the control group. For ^13^C-tracing experiments with [U^13^C]-glutamine and [U^13^C]-lactate, glutamine- and lactate-free EGM-2 medium was supplemented with 10 mM [U^13^C]-glutamine (Cambridge Isotope Laboratories) and 5 mM [U^13^C]-lactate (Sigma-Aldrich), and the ECs were incubated for 48 h to reach steady state. In all experiments, HUVECs and HUAECs were always used as single-donor cultures (represented by n number), were regularly tested for mycoplasma, and were only used between passages (p) 1 and 4.

#### Primary human microvascular dermal endothelial cells

Human microvascular endothelial cells (HMVECs) were freshly isolated from pre-pubertal foreskin biopsies obtained from healthy donors (with approval from the Medical Ethical Committee KU Leuven / UZ Leuven (protocol S57123) and informed consent obtained from all subjects). After dispase digestion to remove the epidermis (overnight incubation at 4°C with 2.5 units/mL dispase II (Sigma-Aldrich)), collagenase digestion (45 min incubation at 37 °C with 0.2% collagenase I solution (Thermo Fisher Scientific)) was performed to harvest all the dermal cells, including ECs. Immunomagnetic beads (Miltenyi Biotec) were used, following the manufacturer’s instructions, to isolate CD45^-^CD31^+^Podoplanin^-^ cells (HMVECs). HMVECs were initially cultured and maintained in Endothelial Cell Growth Medium Microvascular (EGM-MV2), supplemented with the EGM supplement pack (PromoCell)^62^. For experimental procedures ECs were cultured in customized EGM2 medium, as mentioned above for HUVECs and HUAECs. In all experiments, HMVECs were always used as single-donor cultures (represented by n number), were regularly tested for mycoplasma, and were only used between p 1 and 4.

#### Endothelial and cancer cell lines

Murine E2 cells (obtained from E. Dejana^63^) were cultured in Dulbecco’s Modified Eagle Medium (DMEM; Thermo Fisher Scientific) supplemented with 10% FBS, 2 mM glutamine, 100 IU/mL penicillin and 100 µg/mL streptomycin. Liver hepatocellular carcinoma (HepG2) cells were purchased (ATCC) and cultured in DMEM supplemented with 10% FBS, 2 mM L-glutamine, 100 IU/mL penicillin and 100 µg/mL streptomycin. HepG2 cells were used as a positive control for different experiments.

### Knockdown and overexpression strategies

#### Plasmid construction and lentiviral transductions

Plasmid pBABE-puro mCherry-EGFP-LC3B was a gift from Jayanta Debnath^64^, obtained via Addgene (plasmid #22418; http://n2t.net/addgene:22418; RRID:Addgene_22418), from which the puro mCherry-EGFP-LC3B expression cassette was cloned into pRRLsinPPT.CMV.MCS MM WPRE vector. To generate shRNA lentiviral vectors against *PCK2* (two distinct non-overlapping shRNA sequences were used, referred to as KD1 (TRCN0000052666; used for the majority of experiments) and KD2 (TRCN0000052664; used for confirmation of key experiments); Mission shRNA, Merck), oligonucleotides were cloned into the pLKO-shRNA2 vector (No. PT4052-5; Clontech). Empty lentiviral vectors and lentiviral vectors expressing nonsense scrambled shRNA were used as negative controls. All constructs were sequence verified. Production of lentiviruses by transfection into 293 T cells was performed as previously described^65^. For lentiviral transductions, a multiplicity of infection (MOI) of 10 was used for PCK2^KD1^ experiments, 20 was used for PCK2^KD2^ experiments, and 5 was used for mCherry-GFP-LC3 experiments. ECs were transduced overnight and refed with medium the next day. Transduced cells were seeded for experiments at least 4 days post-transduction. Knockdown efficiency was monitored for each experiment at the mRNA (qRT-PCR) and / or protein level.

#### siRNA-mediated gene silencing

Transfection mixtures for negative control siRNA NC5 (10 nM) (IDT Integrated DNA Technologies) and mouse *Pck2*-targeting siRNAs (10 nM) (2 non-overlapping siRNAs were used: siPck2 #1 (mm.Ri.Pck2.13.3) and siPck2 #2 (mm.Ri.Pck2.13.4), IDT Integrated DNA Technologies) were prepared in Opti-MEM with Lipofectamine RNAiMax transfection reagent (Thermo Fisher Scientific) following the manufacturer’s guidelines. The mixtures were added to the cells (150,000 cells in 6-well plate format) together with 2 mL culture medium without antibiotics for overnight transfection after which the transfection medium was replaced with regular culture medium. The cells were further grown for another 24 h, followed by RNA isolation.

### Transcriptomic analysis

#### Bulk RNA-sequencing

RNA of control or PCK2^KD1^ ECs cultured in 5.5 mM *versus* 0 mM glucose conditions was extracted using TRIzol (Thermo Fisher Scientific) following the manufacturer’s instructions. Poly-adenylated fragments were isolated from 1 μg total RNA. These fragments were reverse transcribed and converted into indexed sequencing libraries using the KAPA stranded mRNA-seq kit (Sopachem). The first 50 bases of these libraries were sequenced on a HiSeq 2500 system (Illumina). Raw sequenced reads were aligned to the human reference transcriptome and genome (GRCCh37/hg19) using the Bowtie TopHat pipeline^66^. Mapped reads were assigned to Ensemble gene IDs by HTSeq resulting in on average 26,597,998 ± 6,040,446 assigned reads per sample. After filtering, trimmed mean of M-values (TMM)-normalization was performed using the EdgeR R-package^67^. Heatmaps were created by autoscaling the data gene-wise^68^ and displayed with the heatmaply R-package^69^. Raw RNA-sequencing data are available in the ArrayExpress database (https://www.ebi.ac.uk/arrayexpress/) under accession number ArrayExpress: E-MTAB-8104.

### RNA isolation and quantitative RT-PCR

RNA was collected and purified using the PureLink® RNA Mini Kit (Thermo Fisher Scientific) and converted to cDNA using the iScript cDNA synthesis kit (Bio-Rad) following the manufacturer’s instructions. RNA quality and quantity were measured on a Nanodrop (Thermo Fisher Scientific). mRNA expression analysis was performed by Taqman quantitative (q) RT-PCR (Thermo Fisher Scientific) using premade primer sets (IDT). Human *HPRT1* or mouse *Hprt1* was used as a housekeeping gene. The following premade primer sets were used: *HPRT1* Hs.PT.58.2145446; *Hprt1* Mm.PT.42.12662529; *PCK2* Hs.PT.58.19439369; *Pck2* Mm.PT.58.661.4676; *PCK1* Hs.PT.58.19431010.g; *TJP1* Hs.PT.58.2456962; *CLDN5* Hs.PT.58.1483777.g; *PC* Hs.PT.58.3296529; *FBP* Hs.PT.58.1719755; *PHGDH* Hs.PT.58.2437570; *PSAT1* Hs.PT.58.20540177; *SHMT2* Hs.PT.58.39289648; *GPAM* Hs.PT.58.20958744.

### Biochemical assays

#### Mitochondrial fraction isolation

Mitochondrial fraction isolation was performed using the Mitochondria Isolation Kit for cultured cells (Thermo Fisher Scientific) following the manufacturer’s instructions.

#### Protein extraction

Protein extraction for total cell lysate and immunoblot analysis was performed on ice in RIPA buffer containing protease and phosphatase inhibitors.

#### Bicinchoninic acid (BCA) assay

To determine protein content the Pierce Bicinchoninic Acid (BCA) Protein Assay Kit (Thermo Fisher Scientific) was used following the manufacturer’s instructions.

#### Immunoblot analysis

Protein extracts were separated by SDS-PAGE under reducing conditions, transferred to a nitrocellulose or PVDF membrane, and analyzed by immunoblotting. We used primary antibodies against: PCK2 (D3E11, Cell Signaling Technology #8565, dilution 1:1,000), GAPDH (14C10, Cell Signaling Technology #2118, dilution 1:1,000), LC3B (Abcam #ab51520, dilution 1:3,000), p62/SQSTM1 (Sigma-Aldrich #P0067, dilution 1:1,000), BiP (C50B12, Cell Signaling Technology #3177, dilution 1:1,000), ATF4 (D4B8, Cell Signaling Technology #11815, dilution 1:1,000), CHOP (L63F7, Cell Signaling Technology #2895, dilution 1:1,000), VDAC1 (B-6, Santa Cruz Biotechnology #sc390996, dilution 1:200), SOD1 (Enzo Life Sciences #ADI-SOD-100, dilution 1:1,000), phospho-S6 (Ser235/236) (D57.2.E2, Cell Signaling Technology #4858, dilution 1:2,000), S6 (5G10, Cell Signaling Technology #2217, dilution 1:1,000), phospho-p70S6K (T389) (Cell Signaling Technology #9205, dilution 1:1,000), p70S6K (Cell Signaling Technology #9202, dilution 1:1,000), cleaved ATF6 (Abcam #ab122897, dilution 1:500), spliced XBP1 (XBP1s) (Novus Biologicals #NBP1-77681, dilution 1:1,000), ERp72 (D70D12, Cell Signaling Technology #5033, dilution 1:1,000). GAPDH was used as a loading control. Appropriate HRP-linked secondary antibodies were from Dako. Signal was detected using the ECL system (Amersham Biosciences, GE Healthcare) following the manufacturer’s instructions. Densitometry quantifications of immunoblot bands were done using NIH Image J software.

#### Lactate dehydrogenase (LDH) cell death assay

Cell death was measured by determining the lactate dehydrogenase (LDH) release in the EGM-2 medium using the Cytotoxicity Detection Kit (Roche) following the manufacturer’s instructions. High levels of LDH release signify high cell death and low cell viability.

#### Proteasome activity assay

Proteasome activity was measured in 10,000 ECs per well by determining the Z-nLPnLD (Z-norleucine-proline-norleucine-aspartate)-aminoluciferin luminescence levels emitted upon caspase-like proteolytic activity, using the Proteasome-Glo Caspase-like Cell-Based Assay (Promega) following the manufacturer’s instructions.

### ^13^C Tracer experiments and metabolite level analysis

#### Metabolite pool and isotopomer labeling analysis by LC-MS

For ^13^C-tracing and targeted metabolite measurements of TCA cycle intermediates, aspartate, asparagine, glutamine, glutamate, alanine, histidine, phenylalanine, threonine, tryptophan, tyrosine, methionine, arginine, ornithine, pyruvate, lactate, DHAP, glycerol-3-P, F1,6BP, hexoses (F6P and G6P), ribose-5-P, sedoheptulose-7-P, NADP^+^ and NADPH, ECs (300,000 cells per well in a 6-well plate) were washed with cold 0.9% NaCl solution, snap-frozen in liquid nitrogen and metabolites were extracted with 300 µL ice-cold metabolite extraction buffer (80% methanol containing 2 µM d27 myristic acid). Lysates (supernatant) were collected after centrifugation at 4 °C for 5 min at 20,000 x g and transferred to LC-MS vials, while protein content (pellet) was measured via BCA assay for normalization. Targeted measurements were performed using a Dionex UltiMate 3000 LC System (Thermo Fisher Scientific) coupled to a Q Exactive Orbitrap mass spectrometer (MS) (Thermo Fisher Scientific) operated in negative ion mode. Practically, 35 μL of sample was injected on a SeQuant ZIC/ pHILIC Polymeric column (Merck Millipore). The gradient started with 20% of solvent B (10 mM NH_4_-acetate in MQH_2_O, pH 9.3) and 80% solvent A (LC-MS grade acetonitrile) and remained at 20% B until 2 min post injection. Next, a linear gradient to 80% B was carried out until 29 min. At 38 min the gradient returned to 40% B followed by a decrease to 20% B at 42 min. The chromatography was stopped at 58 min. The flow was kept constant at 100 µL per minute; the column was placed at 25°C throughout the analysis. The MS was operated in full scan (m/z 70–1050) mode using a spray voltage of 3.5 kV, capillary temperature of 320°C, sheath gas at 50.0, auxiliary gas at 10.0. AGC was set at 3e6, maximum IT at 512 ms and a resolution of 140,000. Data collection was performed using Xcalibur software (Thermo Fisher Scientific). For the calculation of the total carbon contribution in ^13^C-tracing experiments we corrected for naturally occurring isotopes using in-house software. For total metabolite levels, the total ion count (arbitrary units of the metabolite of interest) was normalized to the protein content. The total contribution of carbon was calculated using the following equation:

$${{{{{\rm{Total}}}}}}\; {{{{{\rm{contribution}}}}}}\; {{{{{\rm{of}}}}}}\; {{{{{\rm{carbon}}}}}}={\sum }_{i=10}^{n}i* {m}_{i}/\left(n* {\sum }_{i=0}^{n}{m}_{i}\right)$$

Herewith, *n* is the number of C atoms in the metabolite, *i* represents the different mass isotopomers and *m* refers to the abundance of a certain mass.

#### Metabolite pool and isotopomer labeling analysis by GC-MS

For ^13^C-tracing and targeted metabolite measurements of PEP, 2/3-PG, serine and glycine, ECs (300,000 cells per well in a 6-well plate) were washed with cold 0.9% NaCl solution, snap-frozen in liquid nitrogen and metabolites were extracted with 600 µL ice-cold metabolite extraction buffer (80% methanol containing 2 µM d27 myristic acid). Lysates (supernatant) were collected after centrifugation at 4°C for 5 min at 20,000 x g and dried in a Savant Speedvac spd111v (Thermo Fisher Scientific) at 4°C, while protein content (pellet) was measured via BCA assay for normalization. 25 µL of a 2% methoxyamine hydrochloride solution (20 mg dissolved in 1 mL pyridine) was added to the dried fractions which were then incubated at 37°C for 90 min. Then, 75 µL of N-tert-butyldimethylsilyl-N-methyltrifluoroacetamide with 1% N-tertbutyldimethyl-chlorosilane (Sigma-Aldrich) was added and the reaction was carried out for 30 min at 60°C. Reaction mixtures were centrifuged for 15 min at 20,000 x g at 4°C in order to remove insolubilities and the supernatant was transferred to a glass vial with conical insert (Agilent). GC-MS analyses were performed on an Agilent 7890 A GC equipped with a HP-5 ms 5% Phenyl Methyl Silox (30 m - 0.25 mm i.d. - 0.25 µm; Agilent Technologies) capillary column, interfaced with a triple quadrupole tandem mass spectrometer (Agilent 7000B, Agilent Technologies) operating under ionization by electron impact at 70 eV. The injection port, interface and ion source temperatures were kept at 230°C. Temperature of the quadrupoles was kept at 150°C. The injection volume was 1 µL, and samples were injected at 1:5 split ratio. Helium flow was kept constant at 1 mL per minute. The temperature of the column started at 100 °C for 5 min and increased to 260°C, at 2°C per minute. Next, a 40°C per minute gradient was carried out until the temperature reached 300°C. After the gradient, the column was heated for another 3 min at 325°C. The GC-MS analyses were performed in Selected Ion Monitoring (SIM) scanning for the isotopic pattern of metabolites. The calculations for total carbon contribution and total metabolite levels, were performed as described above.

#### Isotopomer labeling analysis in glycerol-phospholipids

For ^13^C-tracing and targeted metabolite measurements of glycerol-phospholipids PC and PE, ECs (1,000,000 cells per 10 cm dish) were washed and collected in 1 mL cold PBS. Cell pellets were resuspended in 800 µL cold PBS, of which 100 µL was used for DNA-determination for normalization. 700 µL of the sample was mixed with 800 µL 1 N HCl:CH_3_OH 1:8 (v/v), 900 µL CHCl_3_ and 200 µg/mL of the antioxidant 2,6-di-tert-butyl-4-methylphenol (BHT; Sigma-Aldrich). The organic fraction was evaporated using a Savant Speedvac spd111v (Thermo Fisher Scientific) at room temperature (RT) and the remaining lipid pellet was stored at -20°C under argon. Prior to mass spectrometry analysis, lipid pellets were reconstituted in 1 mL of CH_3_OH:CHCl_3_ (1:1; v-v). MS analysis was adapted from a previously described protocol^14^. Briefly, measurements were performed using a Dionex UltiMate 3000 LC System (Thermo Fisher Scientific) in-line connected to a Q-Exactive Focus Orbitrap MS (Thermo Fisher Scientific). 10 µL of the sample was injected and loaded onto a Hilicon iHILIC-Fusion(P) column (Achrom). A linear gradient was carried out starting with 90% solvent A (LC-MS grade acetonitrile) and 10% solvent B (10 mM ammoniumacetate pH 9.3). From 2 to 20 min the gradient changed to 80% B and was kept at 80% until 23 min. Next, a decrease to 40% B was carried out to 25 min, further decreasing to 10% B at 27 min. Finally, 10% B was maintained until 35 min. The solvent was used at a flow rate of 200 µL per minute, the column temperature was kept constant at 25°C. The MS was operated in targeted MS2 mode, settings of the HESI probe were as follows: sheath gas flow rate at 35, auxiliary gas flow rate at 10 (at a temperature of 260°C). Spray voltage was set at 4.8 kV, temperature of the capillary at 300°C and S-lens RF level at 50. A targeted MS2 scan was operated as follows: diacyl-PE (nonplasmalogen PE) and plasmalogen-PE molecular species (m/z 745.5 ± 75) and PC molecular species (m/z 804.5 ± 100). For the data analysis we used an in-house library and metabolites of interest were quantified (area under the curve) using the XCalibur 4.0 (Thermo Fisher Scientific) software platform. Correction of natural abundance and calculations of fractional contribution was carried out using an in-house software platform.

#### Phospholipidomics

Lipid composition and characterization was performed as previously described^38^. ECs (1,000,000 cells per 10 cm dish) were washed and collected in 1 mL cold PBS. Cell pellets were resuspended in 800 µL cold PBS, of which 100 µL was used for DNA-determination for normalization. 700 µL of the sample was mixed with 800 µL 1 N HCl:CH_3_OH 1:8 (v/v), 900 µL CHCl_3_ and 200 µg/ml of the antioxidant 2,6-di-tert-butyl-4-methylphenol (BHT; Sigma-Aldrich). The organic fraction was evaporated using a Savant Speedvac spd111v (Thermo Fisher Scientific) at RT and the remaining lipid pellet was stored at -20°C under argon. Prior to mass spectrometry analysis, lipid pellets were reconstituted in running solution (CH_3_OH:CHCl_3_:NH_4_OH; 90:10:1.25; v:v:v). Phospholipid species were analyzed by electrospray ionization tandem mass spectrometry (ESI-MS/MS) on a hybrid triple quadrupole/linear ion trap MS (4000 QTRAP system; Applied Biosystems SCIEX) equipped with a TriVersa NanoMate (Advion Biosciences) robotic nanosource for automated sample injection and spraying. Phospholipid profiling was executed by (positive or negative) precursor ion or neutral loss scanning at a collision energy of 50 eV/45 eV and 35 eV for precursor 184 (sphingomyelin (SM)/phosphatidylcholine (PC)) and neutral loss 141 (phosphatidylethanolamine (PE)), respectively. Phospholipid quantification was performed by multiple reaction monitoring (MRM), the transitions being based on the neutral losses or the typical product ions as described above. The MRM dwell time was set to 100 ms and typically about 20 cycles were used to average the signal. Lipid standards PC25:0, PC43:6, SM30:1, PE25:0 and PE43:6 (Avanti Polar Lipids) were added based on the amount of DNA in the original sample. The data were corrected for isotope effects as previously described^70^. Data was normalized to DNA content.

### Flow cytometry assays

All flow cytometry (FACS) experiments were performed on a BD FACS Aria III and data were analyzed using FlowJo v10.4 software (FlowJo, LLC). Gatings were preformed using appropriate (negative) controls as recommended by the respective assay manufacturer.

#### Total cellular ROS analysis

Intracellular endogenous ROS levels were measured using the 5-(and-6)-chloromethyl-20,70-dichlorodihydrofluorescein diacetate, acetyl ester (CM-H_2_DCFDA) dye (C6827, Thermo Fisher Scientific) following the manufacturer’s instructions. Briefly, prior to the FACS experiment, ECs were incubated for 30 min in 10 µM CM-H_2_DCFDA supplemented serum free EGM-2 medium. Cells were subsequently washed with PBS, harvested via trypsin dissociation and resuspended in PBS + 0.1% FBS.

#### EdU incorporation

Cells in G_0_ were identified as a 2 N DNA containing cell population lacking 5-ethynyl-20-deoxyuridine (EdU) incorporation, while proliferating cells were identified as EdU^+^. Briefly, prior the FACS experiment, cells were labeled with 10 µM EdU over 24 h, washed with PBS, harvested via trypsin dissociation and fixed with 4% paraformaldehyde (PFA). The incorporated EdU was detected by a ‘Click-iT EdU reaction’ with AlexaFluor 647 following the manufacturer’s instructions (Thermo Fisher Scientific).

#### Protein aggregate analysis

Protein aggregate levels were measured using the Proteostat Aggresome detection dye (Enzo Life Sciences ENZ-51035-K100) following the manufacturer’s instructions. The 488 nm excitable red fluorescent molecular rotor dye becomes brightly fluorescent upon binding to aggregated proteins within vesicles produced during aggresome formation. Briefly, prior to the FACS experiment, cells were washed with PBS, harvested via trypsin dissociation and fixed with 4% PFA. Next, cells were permeabilized and stained with 1:10,000 dilution of the Proteostat Aggresome detection dye in PBS + 0.1% FBS for 30 min.

#### Conjugated ubiquitin analysis

Conjugated ubiquitin levels were measured using the (i) mono- and polyubiquitinylated conjugates monoclonal antibody (FK2) (ATTO 488 conjugate) (Enzo Life Sciences BML-PW1335) which recognizes K29-, K48-, and K63-linked polyubiquitinylated and monoubiquitinylated proteins but not free ubiquitin, (ii) K48-specific linkage polyubiquitinylated conjugates monoclonal antibody (Abcam #ab140601) and (iii) K63-specific linkage polyubiquitinylated conjugates monoclonal antibody (Abcam #ab179434). Briefly, prior to the FACS experiment, cells were washed with PBS, harvested via trypsin dissociation and fixed with ice-cold 100% methanol. Next, cells were stained with (i) 1:1,000 dilution of the mono- and polyubiquitinylated conjugates monoclonal antibody (FK2) (ATTO 488 conjugate) in PBS + 0.1% FBS for 2 h or with (ii and iii) first, a 1:100 dilution of the K48- or K63-specific linkage ubiquitin antibody and second, a 1:2,000 dilution of goat anti-rabbit AlexaFluor 488 secondary antibody, in PBS + 0.1% FBS for 30 min each.

#### Autolysosomal degradation

Relative autolysosomal degradation levels were measured as the ratio of median mCherry/GFP fluorescence levels in ECs, as previously described^41,42^, 5 days post-transduction with the mCherry-GFP-LC3 lentiviral vector (MOI 5) and after verification of mCherry and GFP expression by microscopy. By combining the acid-sensitive GFP and the acid-insensitive mCherry, the change from an autophagosome (neutral pH) to autolysosome (acidic pH) can be detected by the specific loss of GFP fluorescence upon acidification of the autophagosome following fusion with the lysosome. Briefly, prior to the FACS experiment, cells were washed with PBS, harvested via trypsin dissociation and resuspended in PBS + 0.1% FBS.

### Functional assays

#### EC spheroid sprouting assay

ECs were incubated overnight in hanging drops in EGM-2 medium containing methylcellulose (20% volume of a 1.2% solution of methylcellulose 4000 cP (Sigma-Aldrich) prepared in EGM-2) to form spheroids. For mitotic inactivation, 1 μg/mL MitoC was added to the medium. Spheroids were then embedded in collagen type 1 (rat tail) gel (Merck Millipore) and cultured for 24 h to induce sprout formation, as previously described^71^. Spheroid cultures were fixed with 4% PFA at RT and imaged under bright field using a Motic AE 31 microscope (Motic Electric Group) or a Leica DMI6000B microscope (Leica Microsystems). Analysis of the number of sprouts and the average sprout length per spheroid was measured using NIH Image J software.

#### Scratch wound migration assay

A scratch wound was applied on confluent EC monolayers (pre-treated with 1 µg/mL MitoC for 16 h) using a 200 µL pipette tip, 24 h after seeding (100,000 cells per well in a 24-well plate). After scratch wounding and photography (T0), the cultures were further incubated in EGM2 medium without MitoC overnight (or until near closure was reached in the control condition) and photographed again (T1). Bright field imaging was done using a Motic AE 31 microscope (Motic Electric Group) or a Leica DMI6000B microscope (Leica Microsystems). Migration (gap area) was measured using NIH Image J software and is expressed as % wound closure according to this formula: 100 − ([gap area at T1]/[gap area at T0]*100).

#### EC proliferation analysis with ^3^H-thymidine

EC proliferation was quantified by incubating the cells with 1 μCi/mL ^3^H-thymidine (Perkin Elmer) for 6 h. Thereafter, cells were washed with PBS, fixed with ice-cold 100% ethanol for 15 min at 4°C, precipitated with 10% trichloroacetic acid and lysed with 0.1 N NaOH. The amount of ^3^H-thymidine incorporated into DNA was measured by scintillation counting.

#### Trans-endothelial electrical resistance assay

The trans-endothelial electrical resistance (TEER) was measured using the Endohm-6 electrode (World Precisions Instruments) connected to an EVOM2 voltohmmeter (World Precisions Instruments). Gelatin-coated transwells without cells were used to measure the intrinsic electrical resistance of the inserts and these values were then subtracted from the values measured in the presence of cells. Measurements were performed on proliferation-blocked confluent EC monolayers (pre-treated with 1 µg/mL MitoC for 16 h), 24 h after seeding (50,000 cells per well seeded in EGM-2 medium on 6.5 mm 0.1% gelatin-coated polyester transwells, 0.4 μm pore size (Costar ref. 3470, Sigma-Aldrich)), and were performed every day for 4 consecutive days, taking at least 2 measurements per well.

### Immunocytochemistry

All methods for histology and immunostainings have been previously described^8,72^. Briefly, unless otherwise stated, ECs were seeded in ibiTreat µ-dishes (ref. 81156, Ibidi), washed with PBS, fixed with 4% PFA for 10 min at RT, and processed for immunocytochemistry. In general, PBS-0.5% Triton + 2% BSA was used to permeabilize the cells, primary antibodies were incubated with the cells overnight at 4 °C, and secondary antibodies were incubated for 2 h at RT. We used primary antibodies against: PCK2 (D3E11, Cell Signaling Technology #8565, dilution 1:200), TOMM20 (Abcam #ab56783, dilution 1:200), CD31 (MEC 13.3, BD Biosciences 557355, dilution 1:200), VE-cadherin (R&D Systems #AF1002, dilution 1:50) and LAMP1 (H4A3, Abcam #ab25630, dilution 1:200). Appropriate fluorescently conjugated secondary antibodies were used: AlexaFluor 488, 568, 633 or 647 (Thermo Fisher Scientific, dilution 1:500). For F-actin staining, a conjugated phalloidin-AlexaFluor 488 probe was used (Thermo Fisher Scientific). For protein aggregate staining, Proteostat Aggresome detection dye was used (Enzo Life Sciences ENZ-51035-K100). Nuclei were counterstained with Hoechst 33258 (1:1000 dilution in PBS) (Sigma-Aldrich). Imaging was done on a Zeiss LSM 780 confocal microscope (oil objectives: x40 with NA 1.3, x63 with NA 1.4, x100 with NA 1.3) (Carl Zeiss). Unless otherwise stated, quantifications were done using NIH Image J software in a minimum of 15 fields per condition per independent experiment (in at least 3 independent experiments).

#### VE-cadherin junction analysis

ECs were seeded in ibiTreat μ-dishes until high confluency was reached. VE-cadherin staining and quantification of junctional length, reticular structures and gap size index were performed as previously described^5^. The total junctional length (100%) was determined by summing up all segments, then the sum of all continuous segments was calculated as the percentage of total junctional length. The percentage difference between total and continuous represents the discontinuous length. Quantification of reticular structures was determined with the formula ([reticular structure area/total cell area] × 1,000)/cell number. Gap size index (intercellular gap area/cell number) was determined with the formula ([intercellular gap area/total cell area] × 1000)/cell number. VE-cadherin staining was also used for the quantification of cell area alone.

#### Apoptosis analysis with (i) TUNEL or (ii) cleaved caspase 3 staining

50,000 ECs per well were seeded in a 24-well plate. ECs present in the supernatant were pooled together with the adherent cells following harvest via trypsin dissociation, then subjected to a centrifuge spin, and resuspended in PBS. From this suspension, aliquots were deposited on microscope slides (Menzel-Gläser Superfrost Plus, Thermo Fisher Scientific) using a cytospin centrifuge (Shandon). The cells were fixed with ice-cold 100% methanol for 10 min and washed twice with PBS. Cells were then: (i) subjected to Terminal dUTP Nick-End Labeling (TUNEL) (Roche) following the manufacturer’s instructions or; (ii) stained for cleaved caspase-3 (rabbit anti cleaved caspase 3, Cell Signaling Technology #9664 S, dilution 1:200). Next, Hoechst 33258 (1:1,000 dilution in PBS) (Sigma-Aldrich) was added to the cells for 10 min at RT to stain the nuclei and the slides were mounted with Pro-Long Gold (Invitrogen, Life Technologies). The percentage of apoptotic cells was determined by counting the (i) TUNEL^+^ cell fraction or the (ii) cleaved caspase-3^+^ fraction in all cells of at least 10 fields per condition per independent experiment (in at least 3 independent experiments).

#### Lipid droplet staining

To determine the presence of intracellular lipid droplets, cells were fixed with 10% formalin for 30 min prior to staining with the Lipid (Oil Red O) Staining Kit (BioVision) following the manufacturer’s instructions.

#### Lysotracker live staining

For live staining of lysosomes (acidic organelles) Lysotracker Red DND-99 (Thermo Fisher Scientific) was used following the manufacturer’s instructions. ECs (100,000 cells seeded per ibiTreat µ-dish) were incubated for 45 min in 50 nM Lysotracker Red DND-99 supplemented EGM-2 medium. Next, Hoechst 33258 (1:1,000 dilution in EGM-2) was added to the cells for 10 min at RT to stain the nuclei. Prior to imaging, ECs were incubated again in fresh EGM-2 medium. Live imaging was done on a Zeiss LSM 780 confocal microscope (oil objectives: x40 with NA 1.3, x63 with NA 1.4, x100 with NA 1.3) (Carl Zeiss).

### Transmission electron microscopy

Transmission electron microscopy (TEM) was performed on a JEOL JEM1400 (JEOL Europe BV) (VIB Bio Imaging Core, Leuven Platform). For TEM observations, samples were fixed for 24 h with 2.5% glutaraldehyde (pH 7.3), buffered with 0.05 M sodium cacodylate. Prior to embedding in Agar 100 Resin (Agar Scientific), the material was post-fixed in 2% OsO_4_ (buffered with 0.05 M sodium cacodylate, pH 7.3) and dehydrated in a graded acetone series. Semi-thin (±1 µm) sections were cut with a Leica Ultracut S microtome and stained with 0.1% thionin - 0.1% methylene blue. The ultra-thin (±70 nm) sections, on copper grids, were stained with uranyl acetate and lead citrate.

### Experimental mouse models

#### Corneal neovascularization

The corneal micropocket assay was performed as previously described^23^. In summary, a lamellar micropocket was dissected (towards the temporal limbus) in the eyes of 8 week-old female C57BL6 mice. In this pocket on the corneal surface, a pellet was inserted containing 80 ng recombinant human basic fibroblast growth factor (bFGF, PeproTech) and 100 ng of a negative control siRNA NC5, of *Pck2*-targeting siRNA mm.Ri.Pck2.13.3 (siPck2 #1) or mm.Ri.Pck2.13.4 (siPck2 #2) (IDT Integrated DNA Technologies). Seven days later, mice were euthanized, eyes were enucleated and corneas were isolated and fixed with ethanol (70%) for whole-mount CD31 antibody staining (primary antibody: rat anti-mouse CD31 MEC 13.3, BD Biosciences 557355, dilution 1:200; secondary antibody: Cy3-donkey anti-rat IgG, Jackson ImmunoResearch 712-165-153, dilution 1:200). After imaging the flat-mounted corneas, the CD31^+^ area was quantified (ImageJ) as percentage of total cornea area. Animal housing and all experimental procedures were approved by the Institutional Animal Ethics Committee of the KU Leuven (Belgium) under protocols P042/2020.

### Statistics and reproducibility

Data represent mean ± s.e.m. of at least three independent experiments, unless otherwise stated. N-numbers, as indicated in the legends to the figures, represent the number of independent experiments performed with freshly isolated HUVECs from individual biological donors (each with at least 3 technical replicates) or the number of individual mice phenotyped. Source data of the quantified data of the main figures are listed in Supplementary Data 1. Statistical significance was calculated by standard two-tailed *t*-test with Welch’s correction, ANOVA (for multiple comparisons (Holm-Šídák test) within one dataset) and by one-sample *t*-test (for comparisons to point-normalized data) using Prism v8.0.0 software (GraphPad). A *P*-value < 0.05 was considered statistically significant.

### Supplementary information

Supplementary Information

Description of additional supplementary files

Supplementary Data 1

## Data Availability

All data generated or analyzed during this study are included in this published article (and its supplementary information files). The raw data that support the findings of this study are available in Supplementary Data 1 or from the corresponding author upon reasonable request. Uncropped and unedited blot images are represented in Supplementary Fig. 10. Raw RNA-sequencing data from control or PCK2-silenced ECs cultured in 5.5 mM *versus* 0 mM glucose conditions are available in the ArrayExpress database (https://www.ebi.ac.uk/arrayexpress/) under accession number ArrayExpress: E-MTAB-8104.
