# Supplementary material for: The gluconeogenesis enzyme PCK2 has a non-enzymatic role in proteostasis in endothelial cells
Source: Commun Biol. 2024 May 23;7:618. doi: 10.1038/s42003-024-06186-6 (PMC11116505; doi:10.1038/s42003-024-06186-6)
Supplement: Supplementary file 1 — Supplementary Information [file 42003_2024_6186_MOESM1_ESM.pdf]

SUPPLEMENTARY INFORMATION

- SUPPLEMENTARY FIGURES
- SUPPLEMENTARY DATA 1 (LEGEND)

Figure S1

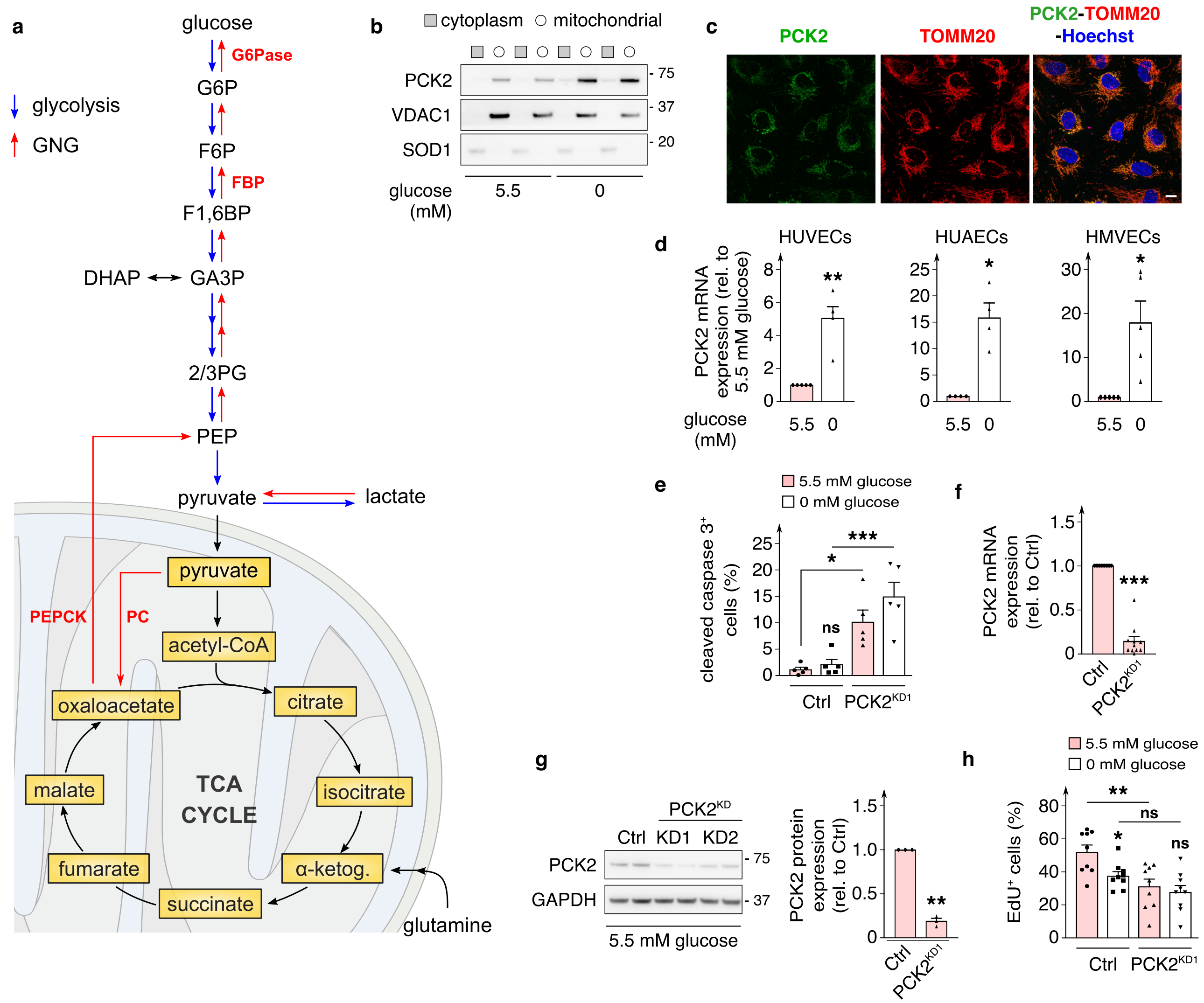
a
glucose
G6Pase
G6P
glycolysis
GNG
F6P
FBP
F1,6BP
DHAP
GA3P
2/3PG
PEP
pyruvate
lactate
pyruvate
PEPCK
PC
acetyl-CoA
oxaloacetate
citrate
malate
TCA CYCLE
isocitrate
fumarate
α-ketog.
succinate
glutamine
b
cytoplasm
mitochondrial
PCK2
VDAC1
SOD1
75
37
20
glucose (mM)
5.5
0
c
PCK2
TOMM20
PCK2-TOMM20-Hoechst
d
HUVECs
HUAECs
HMVECs
PCK2 mRNA expression (rel. to 5.5 mM glucose)
glucose (mM)
e
5.5 mM glucose
0 mM glucose
cleaved caspase 3+ cells (%)
ns
Ctrl
PCK2KD1
f
PCK2 mRNA expression (rel. to Ctrl)
Ctrl
PCK2KD1
g
PCK2KD
Ctrl
KD1
KD2
PCK2
GAPDH
5.5 mM glucose
PCK2 protein expression (rel. to Ctrl)
h
5.5 mM glucose
0 mM glucose
EdU+ cells (%)
ns
Ctrl
PCK2KD1

SUPPLEMENTARY FIGURE 1: ROLE OF ENDOTHELIAL PCK2 IN VESSEL SPROUTING

a, Graphical representation of the gluconeogenic (GNG) and glycolytic pathway with indication of the key rate-controlling GNG enzymes glucose-6-phosphatase (G6Pase), fructose-1,6-bisphosphatase (FBP), phosphoenol-pyruvate (PEP) carboxykinase (PEPCK, also termed PCK) and pyruvate carboxylase (PC). Other abbreviations (from top to bottom): G6P, glucose-6-phosphate; F6P, fructose-6-phosphate; F1,6BP, fructose-1,6-bisphosphate; GA3P, glyceraldehyde-3-phosphate; DHAP, dihydroxyacetone phosphate; 2/3PG, 2/3-phosphoglycerate; α-ketog., α-ketoglutarate; TCA cycle, tricarboxylic acid cycle. **b,** Representative immunoblot of PCK2 protein levels in mitochondrial and cytoplasmic fractions of ECs cultured in 5.5 *versus* 0 mM glucose (n=3). VDAC1 is used as a mitochondrial marker; SOD1 is used as a cytoplasmic marker. Squares: cytoplasm; circles: mitochondrial. **c,** Representative immunofluorescence images of PCK2 (green), TOMM20 (red; mitochondrial marker) and Hoechst (blue; nucleus marker) staining in ECs cultured in 5.5 mM glucose (scale bar represents 10 µm) (representative for n=3 independent experiments). **d,** qRT-PCR analysis of *PCK2* mRNA level in human venous ECs (HUVECs; n=5), arterial ECs (HUAECs; n=4) and microvascular ECs (HMVECs; n=5) cultured for 48 hours in 5.5 *versus* 0 mM glucose (data expressed relative to 5.5 mM glucose). **e,** Quantification of apoptotic cleaved caspase 3^{+} in control and $PCK2^{KD1}$ ECs in 5.5 *versus* 0 mM glucose (n=5). **f,** qRT-PCR analysis of *PCK2* mRNA level in control and $PCK2^{KD1}$ ECs in 5.5 mM glucose (data expressed relative to control (Ctrl)) (n=11). **g,** Representative immunoblot and densitometric quantification of PCK2 protein level in Ctrl, $PCK2^{KD1}$ (n=3) and $PCK2^{KD2}$ (n=6) ECs in 5.5 mM glucose (densitometric data relative to Ctrl). GAPDH was used as a loading control. **h,** EdU incorporation into DNA in control and $PCK2^{KD1}$ ECs in 5.5 *versus* 0 mM glucose (n=9). Data are mean ± s.e.m. Statistics: one-sample t-test (**d,f,g**), ANOVA (**e,h**); * $P< 0.05$; ** $P< 0.01$; *** $P< 0.001$; ns, not significant.

Figure S2

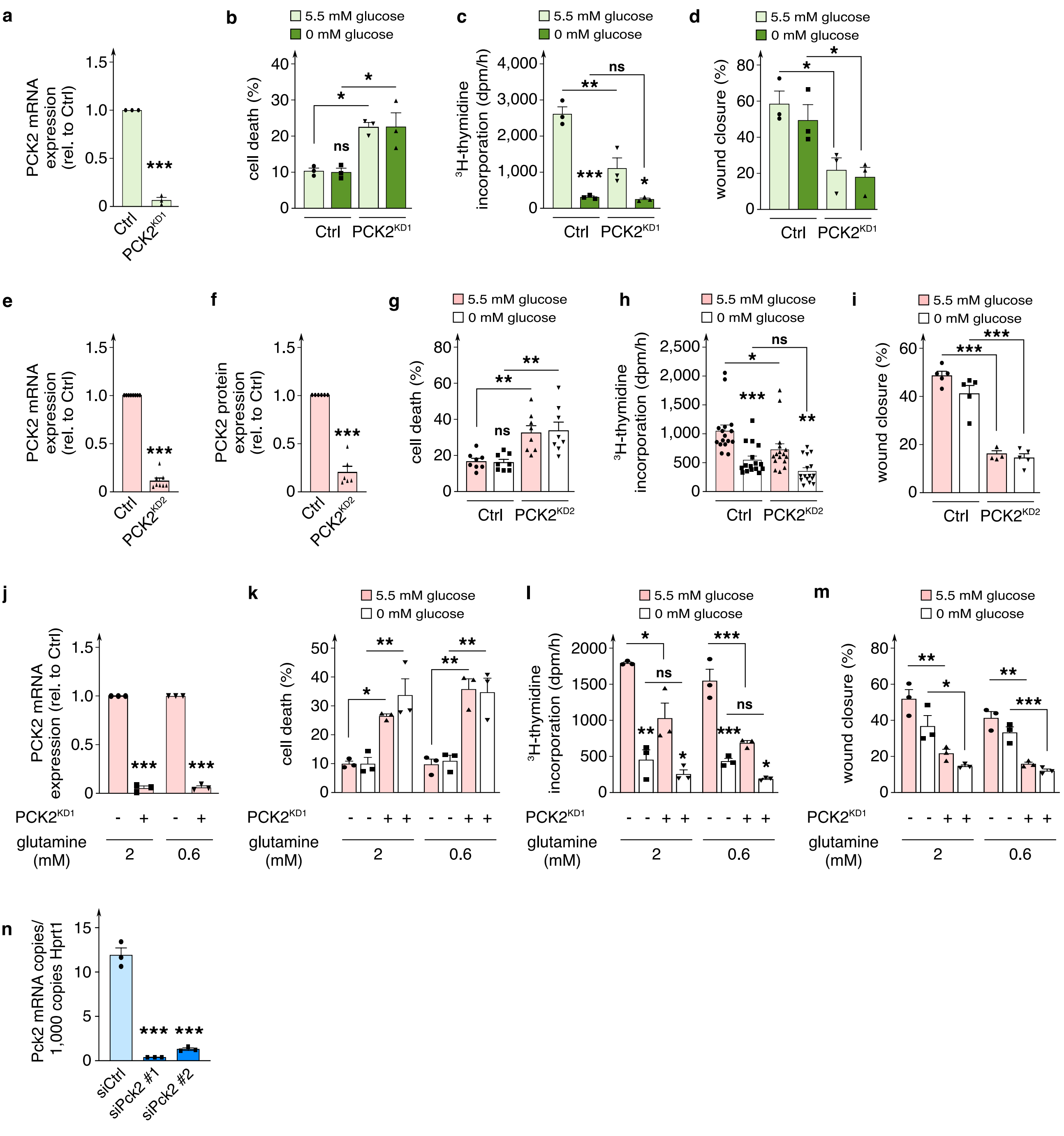

a
PCK2 mRNA expression (rel. to Ctrl)
Ctrl
PCK2KD1
b
5.5 mM glucose
0 mM glucose
cell death (%)
c
3H-thymidine incorporation (dpm/h)
d
wound closure (%)
e
PCK2KD2
f
PCK2 protein expression (rel. to Ctrl)
g
h
i
j
glutamine (mM)
k
l
m
n
Pck2 mRNA copies/ 1,000 copies Hprt1
siCtrl
siPck2 #1
siPck2 #2

Supplementary figure 2: Silencing of *PCK2* in different EC types and conditions

a, qRT-PCR analysis of *PCK2* mRNA level in control and $PCK2^{KD1}$ HUAECs in 5.5 mM glucose (data expressed relative to control (Ctrl)) (n=3). **b,c,** Cell death (**b**) and ^{3}H-thymidine incorporation into DNA (**c**) in control and $PCK2^{KD1}$ HUAECs in 5.5 *versus* 0 mM glucose (n=3). **d,** Scratch wound closure in MitoC-treated control and $PCK2^{KD1}$ HUAECs in 5.5 *versus* 0 mM glucose (n=3). **e,** qRT-PCR analysis of *PCK2* mRNA level in control and $PCK2^{KD2}$ ECs in 5.5 mM glucose (data expressed relative to control (Ctrl)) (n=8). **f,** Densitometric quantification of PCK2 protein level in Ctrl and $PCK2^{KD2}$ ECs in 5.5 mM glucose (densitometric data relative to Ctrl) (n=6). See also representative immunoblot in Supplementary Figure 1g. **g,h,** Cell death (**g**; n=8) and ^{3}H-thymidine incorporation into DNA (**h**; n=16) in control and $PCK2^{KD2}$ ECs in 5.5 *versus* 0 mM glucose. **i,** Scratch wound closure in MitoC-treated control and $PCK2^{KD2}$ ECs in 5.5 *versus* 0 mM glucose (n=5). **j,** qRT-PCR analysis of *PCK2* mRNA level in control and $PCK2^{KD1}$ ECs in 5.5 mM glucose in lower glutamine concentrations (2 or 0.6 mM; data expressed relative to control (Ctrl)) (n=3). **k,l,** Cell death (**k**) and ^{3}H-thymidine incorporation into DNA (**l**) in control and $PCK2^{KD1}$ ECs in 5.5 *versus* 0 mM glucose in lower glutamine concentrations (2 or 0.6 mM) (n=3). **m,** Scratch wound closure in MitoC-treated control and $PCK2^{KD1}$ ECs in 5.5 *versus* 0 mM glucose in lower glutamine concentrations (2 or 0.6 mM) (n=3). **n,** qRT-PCR analysis of *Pck2* mRNA level in murine E2 ECs transfected with negative control siRNA (siCtrl) or siRNAs against murine *Pck2*, expressed relative to the *Hprt1* housekeeping gene (n=3). Data are mean ± s.e.m. Statistics: one-sample t-test (**a,e,f,j**), ANOVA (**b-d,g-I,k-n**); * *P*< 0.05; ** *P*< 0.01; *** *P*< 0.001; ns, not significant. dpm, disintegrations per minute.

Figure S3

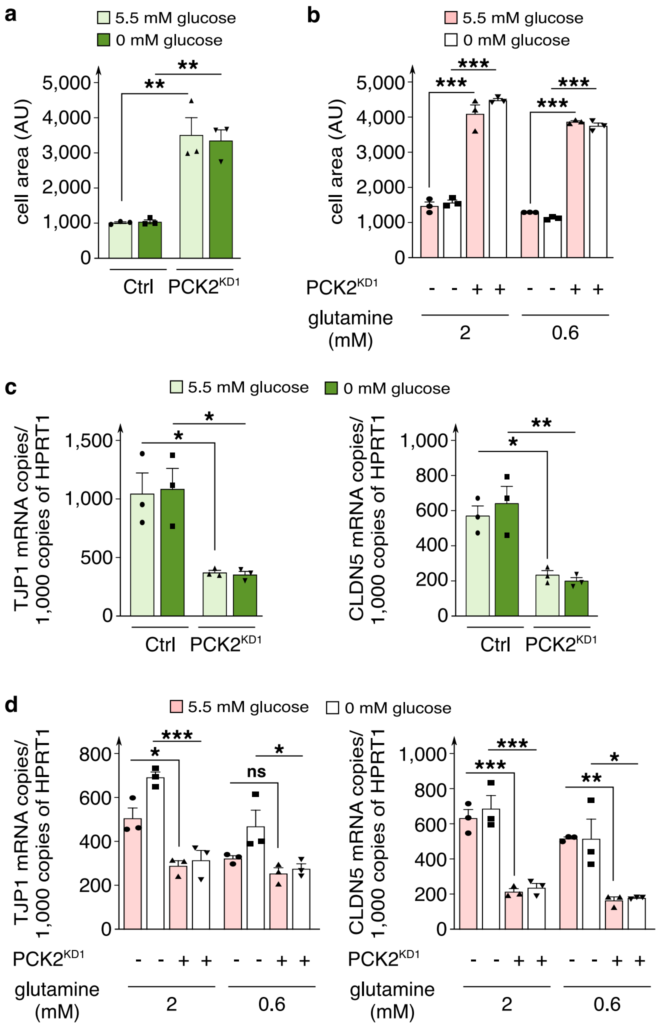

a
5.5 mM glucose
0 mM glucose
cell area (AU)
5,000
4,000
3,000
2,000
1,000
0
**
**
Ctrl
PCK2KD1
b
5.5 mM glucose
0 mM glucose
cell area (AU)
***
***
***
***
PCK2KD1
- - + + - - + +
glutamine (mM)
2
0.6
c
5.5 mM glucose
0 mM glucose
TJP1 mRNA copies/ 1,000 copies of HPRT1
1,500
1,000
500
0
*
*
Ctrl
PCK2KD1
CLDN5 mRNA copies/ 1,000 copies of HPRT1
1,000
800
600
400
200
0
*
**
Ctrl
PCK2KD1
d
5.5 mM glucose
0 mM glucose
TJP1 mRNA copies/ 1,000 copies of HPRT1
800
600
400
200
0
*
***
ns
*
PCK2KD1
- - + + - - + +
glutamine (mM)
2
0.6
CLDN5 mRNA copies/ 1,000 copies of HPRT1
1,000
800
600
400
200
0
***
***
**
*
PCK2KD1
- - + + - - + +
glutamine (mM)
2
0.6

Supplementary figure 3: Effect of PCK2 silencing on EC morphology and membrane integrity

a, Quantification of cell area in control and PCK2^{KD1} HUAECs in 5.5 *versus* 0 mM glucose (n=3). **b,** Quantification of cell area in control and PCK2^{KD1} ECs in 5.5 *versus* 0 mM glucose in lower glutamine concentrations (2 or 0.6 mM) (n=3). **c,** qRT-PCR analysis of tight junction protein 1 (*TJP1*) and claudin-5 (*CLDN5*) mRNA levels in control and PCK2^{KD1} HUAECs in 5.5 *versus* 0 mM glucose (n=3). **d**, qRT-PCR analysis of tight junction protein 1 (*TJP1*) and claudin-5 (*CLDN5*) mRNA levels in control and PCK2^{KD1} ECs in 5.5 *versus* 0 mM glucose in lower glutamine concentrations (2 or 0.6 mM) (n=3). Data are mean ± s.e.m. Statistics: ANOVA (**a-d**); * $P< 0.05$; ** $P< 0.01$; *** $P< 0.001$; ns, not significant. AU, arbitrary units.

Figure S4

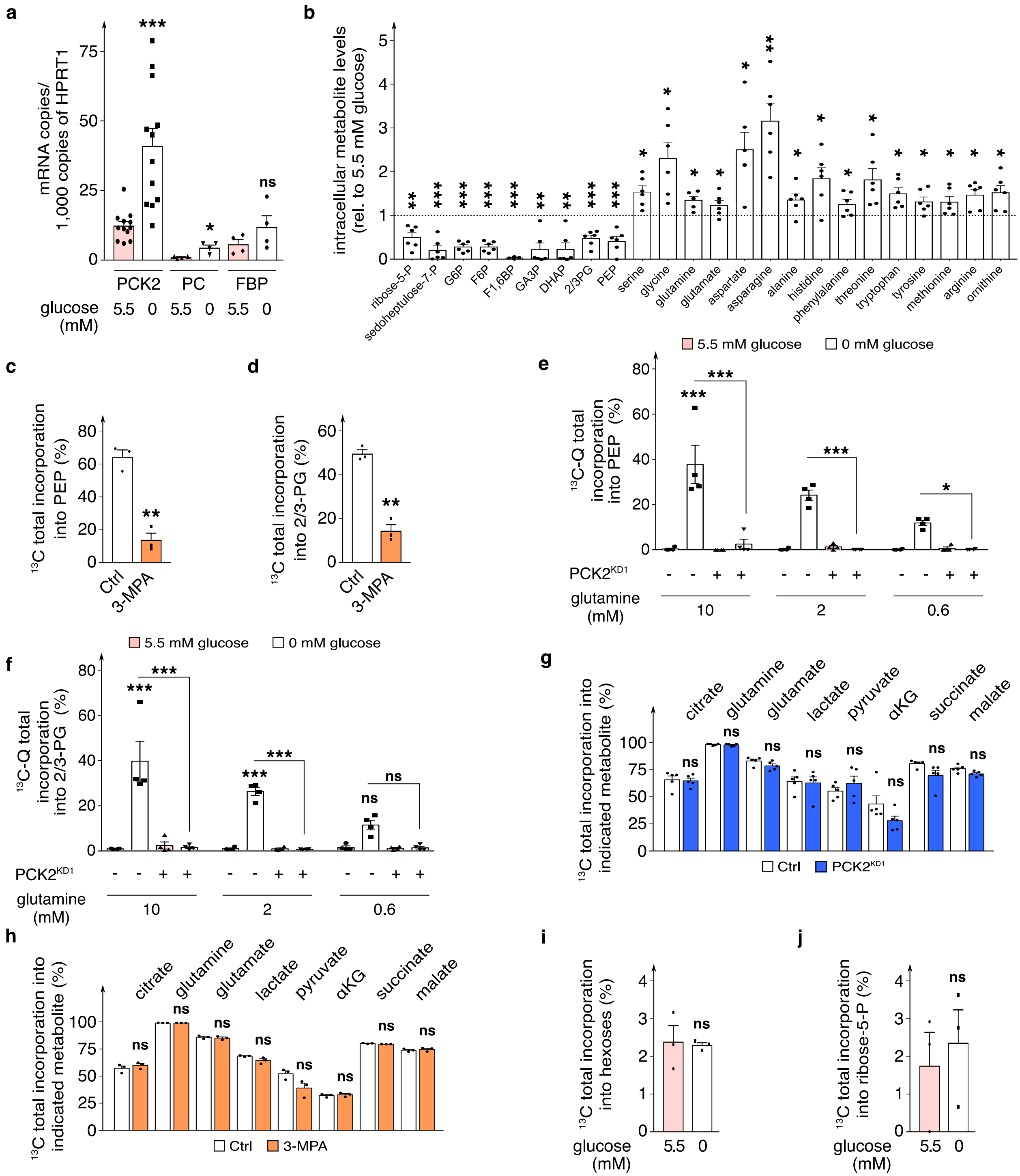
a
mRNA copies/ 1,000 copies of HPRT1
PCK2
PC
FBP
glucose (mM)
5.5
0
ns
b
intracellular metabolite levels (rel. to 5.5 mM glucose)
ribose-5-P
sedoheptulose-7-P
G6P
F6P
F1,6BP
GA3P
DHAP
2/3PG
PEP
serine
glycine
glutamine
glutamate
aspartate
asparagine
alanine
histidine
phenylalanine
threonine
tryptophan
tyrosine
methionine
arginine
ornithine
5.5 mM glucose
0 mM glucose
c
13C total incorporation into PEP (%)
Ctrl
3-MPA
d
13C total incorporation into 2/3-PG (%)
e
13C-Q total incorporation into PEP (%)
PCK2KD1
glutamine (mM)
10
2
0.6
f
13C-Q total incorporation into 2/3-PG (%)
g
13C total incorporation into indicated metabolite (%)
citrate
glutamine
glutamate
lactate
pyruvate
αKG
succinate
malate
Ctrl
PCK2KD1
h
3-MPA
i
13C total incorporation into hexoses (%)
j
13C total incorporation into ribose-5-P (%)

Supplementary figure 4: PCK2 controls channeling of glutamine- and lactate-derived carbons into lower glycolytic intermediates in glucose-deprived ECs

a, qRT-PCR analysis of *PCK2* (n=12), *PC* (n=4) and *FBP* (n=4) mRNA levels in ECs cultured in 5.5 *versus* 0 mM glucose. **b,** Intracellular levels of pentose phosphate pathway metabolites (ribose-5-phosphate and sedoheptulose-7-phosphate), glycolytic intermediates and amino acids in glucose-deprived ECs (relative to control 5.5 mM glucose ECs; represented by the dotted line) (n=6). **c,d,** Incorporation of [$U^{13}C$]-glutamine and [$U^{13}C$]-lactate carbon into total intracellular PEP pool (**c**) and 2/3-PG pool (**d**) in control and 3-mercaptopicolinic acid (3-MPA; 100 µM, 48 hour)-treated glucose-deprived ECs (n=3). **e,f,** Incorporation of [$U^{13}C$]-glutamine carbon into total intracellular phosphoenolpyruvate (PEP) pool (**e**) and 2/3-phosphoglycerate (2/3-PG) pool (**f**) in control and $PCK2^{KD1}$ ECs in 5.5 *versus* 0 mM glucose in different glutamine concentrations (10, 2 or 0.6 mM) (n=3). **g,h,** Incorporation of [$U^{13}C$]-glutamine and [$U^{13}C$]-lactate carbon into total intracellular pool of metabolites downstream of PEP (including TCA cycle intermediates) in control and $PCK2^{KD1}$ (**g**; n=5) or 3-MPA-treated (**h**; n=3) glucose-deprived ECs. **i,j,** Incorporation of [$U^{13}C$]-glutamine and [$U^{13}C$]-lactate carbon into total intracellular hexoses (fructose-6-phosphate (F6P) and glucose-6-phosphate (G6P)) pool (**i**) and ribose-5-phosphate pool (**j**) in ECs in 5.5 *versus* 0 mM glucose (n=3). Data are mean ± s.e.m. Statistics: two-tailed t-test with Welch's correction (**a,c,d,g-j**), ANOVA (**e,f**), one-sample t-test (**b**); * $P< 0.05$; ** $P< 0.01$; *** $P< 0.001$; ns, not significant. αKG, α-ketoglutarate.

Figure S5

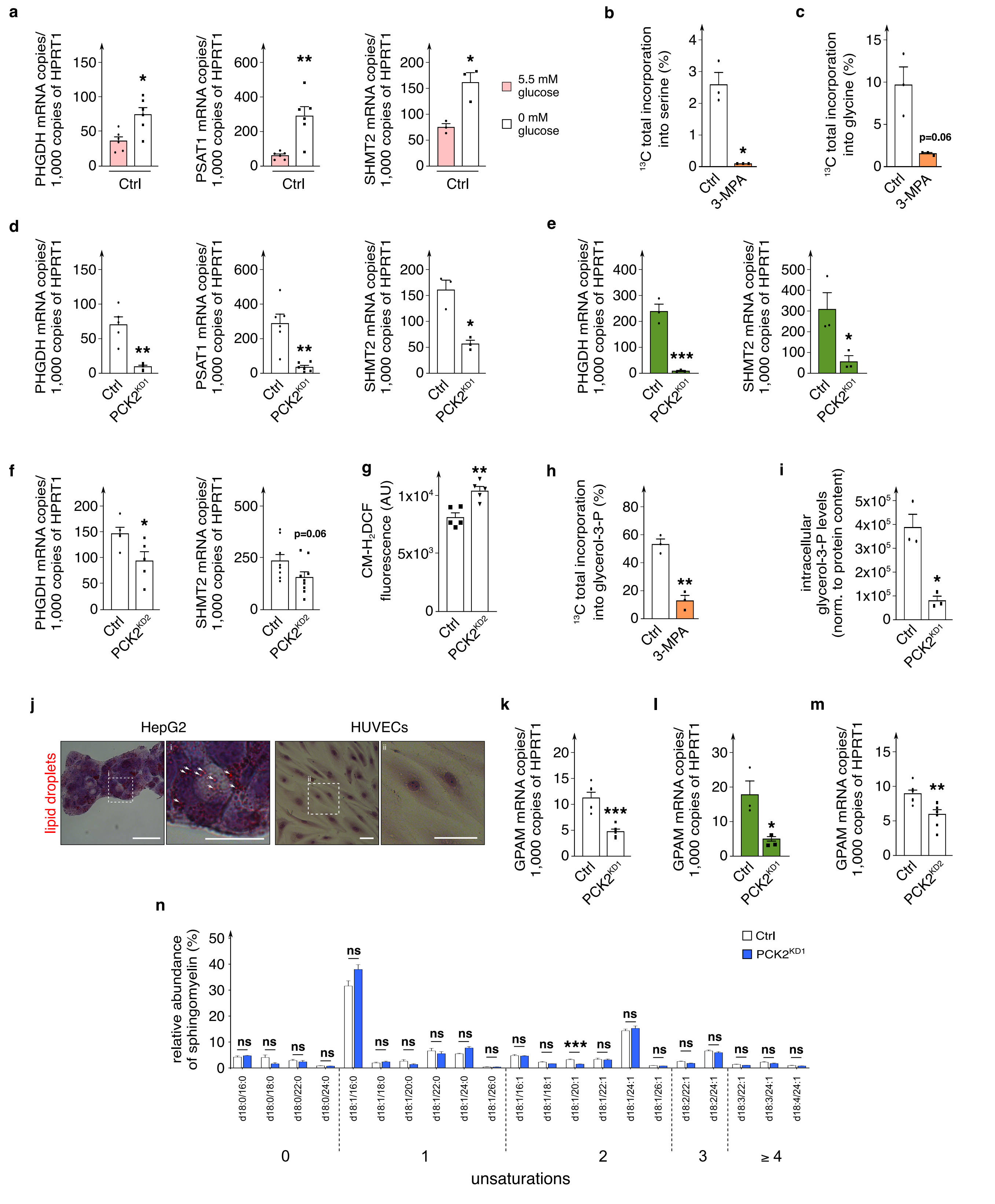

a
PHGDH mRNA copies/ 1,000 copies of HPRT1
PSAT1 mRNA copies/ 1,000 copies of HPRT1
SHMT2 mRNA copies/ 1,000 copies of HPRT1
Ctrl
5.5 mM glucose
0 mM glucose
b
13C total incorporation into serine (%)
c
13C total incorporation into glycine (%)
p=0.06
Ctrl
3-MPA
d
e
PCK2KD1
f
PCK2KD2
p=0.06
g
CM-H2DCF fluorescence (AU)
h
13C total incorporation into glycerol-3-P (%)
i
intracellular glycerol-3-P levels (norm. to protein content)
j
HepG2
HUVECs
lipid droplets
k
l
m
GPAM mRNA copies/ 1,000 copies of HPRT1
n
relative abundance of sphingomyelin (%)
Ctrl
PCK2KD1
d18:0/16:0
d18:0/18:0
d18:0/22:0
d18:0/24:0
d18:1/16:0
d18:1/18:0
d18:1/20:0
d18:1/22:0
d18:1/24:0
d18:1/26:0
d18:1/16:1
d18:1/18:1
d18:1/20:1
d18:1/22:1
d18:1/24:1
d18:1/26:1
d18:2/22:1
d18:2/24:1
d18:3/22:1
d18:3/24:1
d18:4/24:1
0
1
2
3
≥ 4
unsaturations

SUPPLEMENTARY FIGURE 5: PCK2-DERIVED GLYCOLYTIC INTERMEDIATES SHUTTLE INTO SERINE/GLYCINE BIOSYNTHESIS AND GLYCERONEOGENESIS

a, qRT-PCR analysis of *PHGDH* (n=6), *PSAT1* (n=6) and *SHMT2* (n=3) mRNA levels in ECs cultured in 5.5 *versus* 0 mM glucose. **b,c,** Incorporation of [$U^{13}C$]-glutamine and [$U^{13}C$]-lactate carbon into total intracellular serine pool (**b**) and glycine pool (**c**) in control or 3-MPA-treated glucose-deprived ECs (n=3). **d-f,** qRT-PCR analysis of mRNA levels of genes involved in serine and glycine biosynthesis pathway in control and $PCK2^{KD1}$ glucose-deprived ECs (**d**; *PHGDH*, n=6; *PSAT1*, n=6; *SHMT2*, n=3), in control and $PCK2^{KD1}$ glucose-deprived HUAECs (**e**; *PHGDH*, n=3; *SHMT2*, n=3), or in control and $PCK2^{KD2}$ glucose-deprived ECs (**f**; *PHGDH*, n=5; *SHMT2*, n=9). **g,** Quantification of cellular ROS levels (measured as median CM-H_2DCF fluorescence levels) in control and $PCK2^{KD2}$ glucose-deprived ECs (n=3). **h,** Incorporation of [$U^{13}C$]-glutamine and [$U^{13}C$]-lactate carbon into total intracellular glycerol-3-phosphate pool in control and 3-MPA-treated glucose-deprived ECs (n=3). **i,** Intracellular levels of glycerol-3-phosphate, normalized (norm.) to protein content (expressed in AUC/µg protein), in control and $PCK2^{KD1}$ glucose-deprived ECs (n=3). **j,** Representative images of lipid droplet staining in HepG2 cells (used as positive control) and HUVECs. For both cell types, the panels to the right are magnifications of the respective boxed area indicated in the left panel (scale bars represent 50 µm in the original left panels, 25 µm in the magnifications). White arrowheads denote the presence of lipid droplets (red) (representative for n=3 independent experiments). **k-m,** qRT-PCR analysis of *GPAM* mRNA levels in control and $PCK2^{KD1}$ glucose-deprived ECs (**k**; n=6), in control and $PCK2^{KD1}$ glucose-deprived HUAECs (**l**; n=3), or in control and $PCK2^{KD2}$ glucose-deprived ECs (**m**; n=7). **n,** Phospholipidomic profile analysis showing the relative abundance of saturated, mono-, di- and poly-unsaturated sphingomyelin lipid species in control or $PCK2^{KD1}$ glucose-deprived ECs (n=4). The numbers under the bars indicate the carbon length of the fatty acid for the different sphingomyelin lipid species within each group of unsaturation degree (zero to $\geq$ 4). Data are mean ± s.e.m. Statistics: two-tailed t-test with Welch's correction (**a-i,k-n**); * $P< 0.05$; ** $P< 0.01$; *** $P< 0.001$; ns, not significant. AU, arbitrary units.

Figure S6

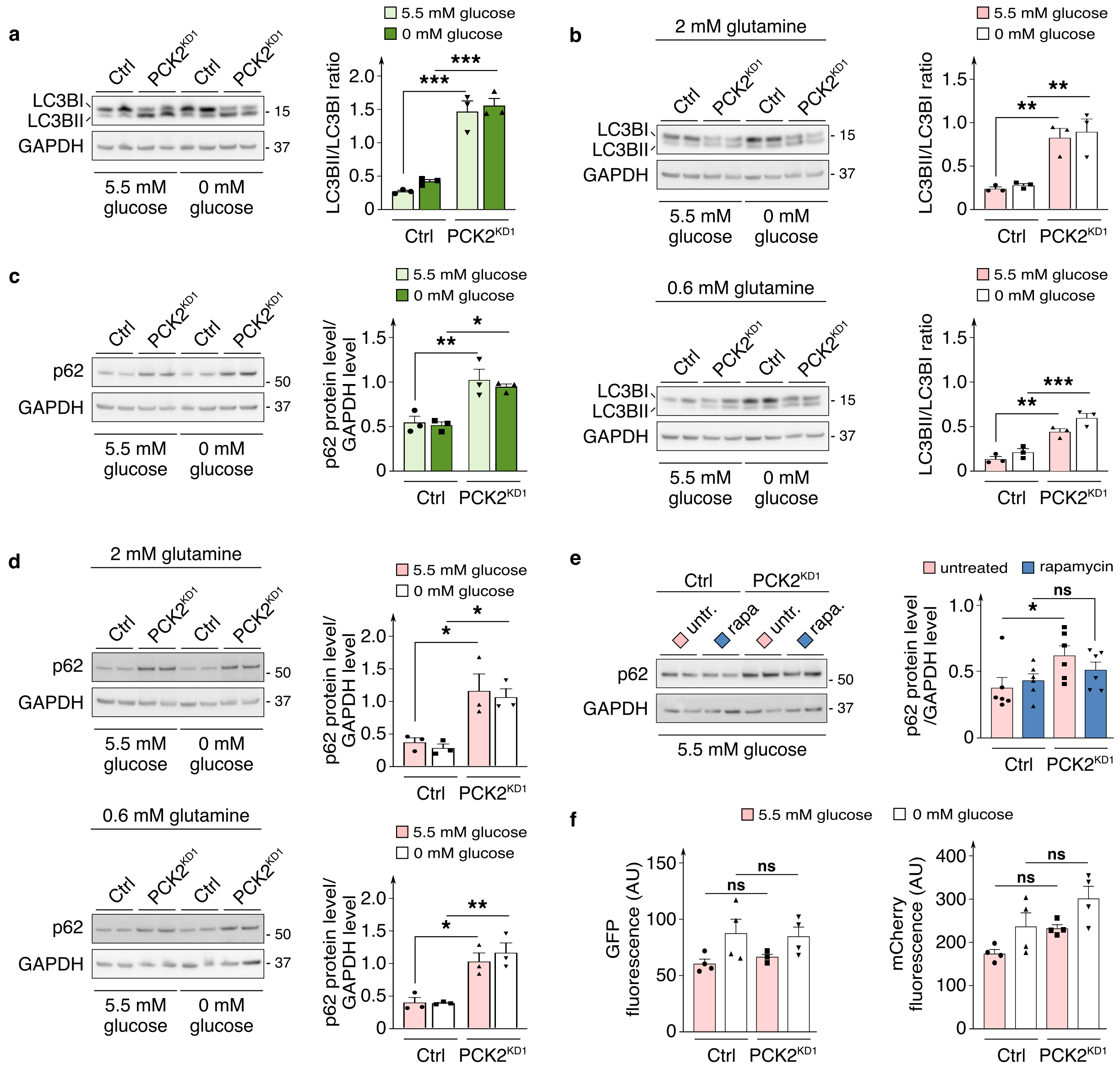

a
Ctrl
PCK2KD1
LC3BI
LC3BII
- 15
GAPDH
- 37
5.5 mM glucose
0 mM glucose
LC3BII/LC3BI ratio
Ctrl
b
2 mM glutamine
0.6 mM glutamine
c
p62
- 50
p62 protein level/ GAPDH level
d
e
untr.
rapa
untreated
rapamycin
ns
p62 protein level /GAPDH level
f
GFP fluorescence (AU)
mCherry fluorescence (AU)

Supplementary figure 6: PCK2-silenced ECs have decelerated autolysosomal clearance of protein aggregates

a,b, Representative immunoblot of LC3B protein level and densitometric quantification of LC3BII (lipidated)/LC3BI (non-lipidated) ratio in control and PCK2^{KD1} HUAECs in 5.5 *versus* 0 mM glucose (**a**; n=3) or in control and PCK2^{KD1} ECs in 5.5 *versus* 0 mM glucose in lower glutamine concentrations (2 or 0.6 mM) (**b**; n=3). GAPDH was used as a loading control. **c,d,** Representative immunoblot and densitometric quantification of p62 protein level in control and PCK2^{KD1} HUAECs in 5.5 *versus* 0 mM glucose (**c**; n=3) or in control and PCK2^{KD1} ECs in 5.5 *versus* 0 mM glucose in lower glutamine concentrations (2 or 0.6 mM) (**d**; n=3). GAPDH was used as a loading control. Immunoblotting for p62 in ECs was done on the same blot as the LC3BI/II immunoblotting of panel b (see above), hence the same GAPDH loading control is shown in both panels. **e,** Representative immunoblot and densitometric quantification of p62 protein levels in control and PCK2^{KD1} ECs in 5.5 mM glucose with 24-hour 50 nM rapamycin (rapa.) or without treatment (untr.) (n=6). **f,** Quantification of median GFP and mCherry fluorescence levels in control and PCK2^{KD1} ECs overexpressing mCherry(acid-insensitive)-GFP(acid-sensitive)-LC3 (see Methods) in 5.5 *versus* 0 mM glucose (n=4). Data are mean ± s.e.m. Statistics: ANOVA (**a-f**); * $P< 0.05$; ** $P<0.01$; *** $P< 0.001$; ns, not significant. AU, arbitrary units.

Figure S7

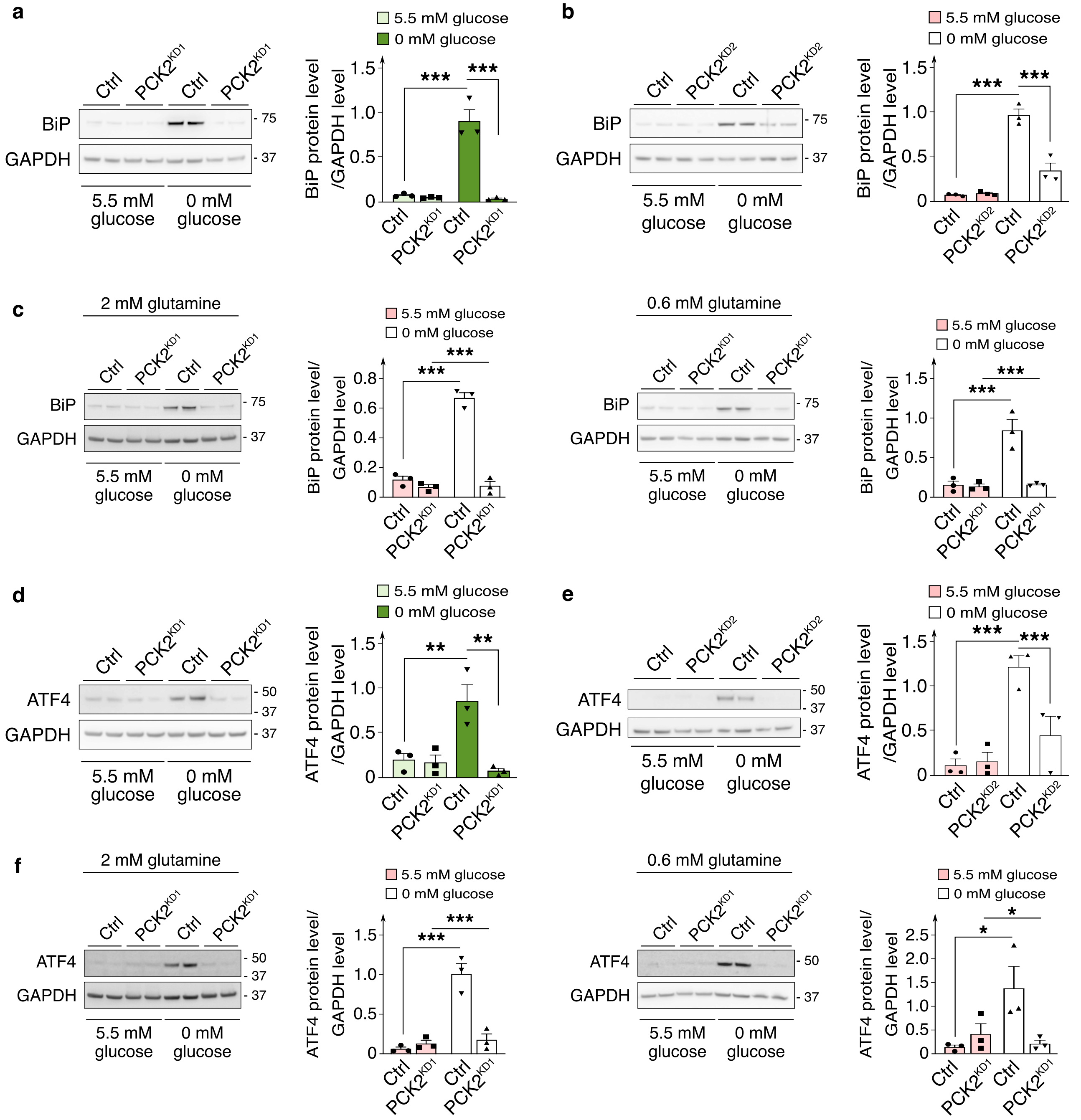
a
5.5 mM glucose
0 mM glucose
Ctrl
PCK2KD1
BiP
GAPDH
75
37
5.5 mM glucose
0 mM glucose
BiP protein level /GAPDH level
***
b
PCK2KD2
c
2 mM glutamine
0.6 mM glutamine
BiP protein level/ GAPDH level
d
ATF4
50
ATF4 protein level /GAPDH level
**
e
f
ATF4 protein level/ GAPDH level
*

SUPPLEMENTARY FIGURE 7: EFFECT OF PCK2 SILENCING ON PROTEOSTASIS IN GLUCOSE-DEPRIVED ECS

a-c Representative immunoblot and densitometric quantification of BiP protein levels in control and PCK2^{KD1} HUAECs in 5.5 *versus* 0 mM glucose (**a**; n=3), in control and PCK2^{KD2} ECs in 5.5 *versus* 0 mM glucose (**b**; n=3), or in control and PCK2^{KD1} ECs in 5.5 *versus* 0 mM glucose in lower glutamine concentrations (2 or 0.6 mM) (**c**; n=3). GAPDH was used as a loading control. **d-f** Representative immunoblot and densitometric quantification of ATF4 protein levels in control and PCK2^{KD1} HUAECs in 5.5 *versus* 0 mM glucose (**d**; n=3), in control and PCK2^{KD2} ECs in 5.5 *versus* 0 mM glucose (**e**; n=3), or in control and PCK2^{KD1} ECs in 5.5 *versus* 0 mM glucose in lower glutamine concentrations (2 or 0.6 mM) (**f**; n=3). GAPDH was used as a loading control. Immunoblotting of ECs in f (2 mM glutamine condition) was done on the same blot as the BiP immunoblotting of panel c (2 mM condition) (see above), hence the same GAPDH loading control is shown in both panels. Data are mean ± s.e.m. Statistics: ANOVA (**a-f**); * $P< 0.05$; ** $P<0.01$; *** $P< 0.001$; ns, not significant.

Figure S8

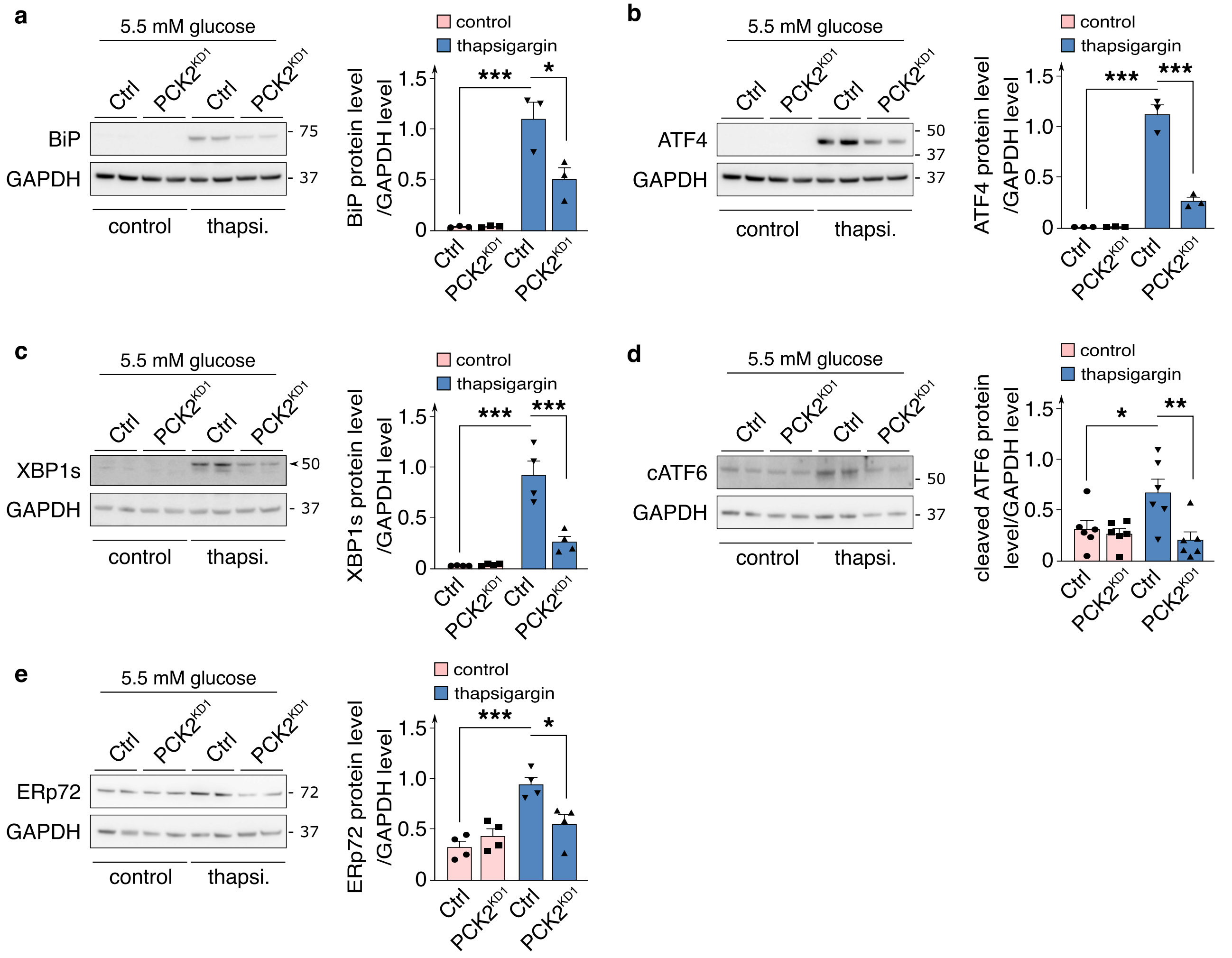
a
5.5 mM glucose
Ctrl
PCK2KD1
Ctrl
PCK2KD1
BiP
GAPDH
75
37
control
thapsi.
control
thapsigargin
BiP protein level /GAPDH level
1.5
1.0
0.5
0
***
*
b
5.5 mM glucose
ATF4
GAPDH
50
37
37
control
thapsi.
ATF4 protein level /GAPDH level
***
***
c
5.5 mM glucose
XBP1s
GAPDH
50
37
control
thapsi.
XBP1s protein level /GAPDH level
***
***
d
5.5 mM glucose
cATF6
GAPDH
50
37
control
thapsi.
cleaved ATF6 protein level/GAPDH level
*
**
e
5.5 mM glucose
ERp72
GAPDH
72
37
control
thapsi.
ERp72 protein level /GAPDH level
***
*

Supplementary figure 8: Effect of PCK2 silencing on proteostasis in normal glucose ECs upon treatment with ER stressor (thapsigargin)

a-e, Representative immunoblot and densitometric quantification of BiP (**a**; n=3), ATF4 (**b**; n=3), spliced XBP1 (XBP1s; see black arrowhead) (**c**; n=4), cleaved ATF6 (cATF6) (**d**; n=6) and ERp72 (**e**; n=4) protein levels in control and PCK2^{KD1} ECs in 5.5 mM glucose with 6-hour 2 µM thapsigargin (thapsi.) or without treatment (control). GAPDH was used as a loading control. Immunoblotting for ATF4 (panel b) was done on the same blot as the immunoblotting of BiP (panel a), hence the same GAPDH loading control is shown in both panels. Data are mean ± s.e.m. Statistics: ANOVA (**a-e**); * *P*< 0.05; ** *P*< 0.01; *** *P*< 0.001.

Figure S9

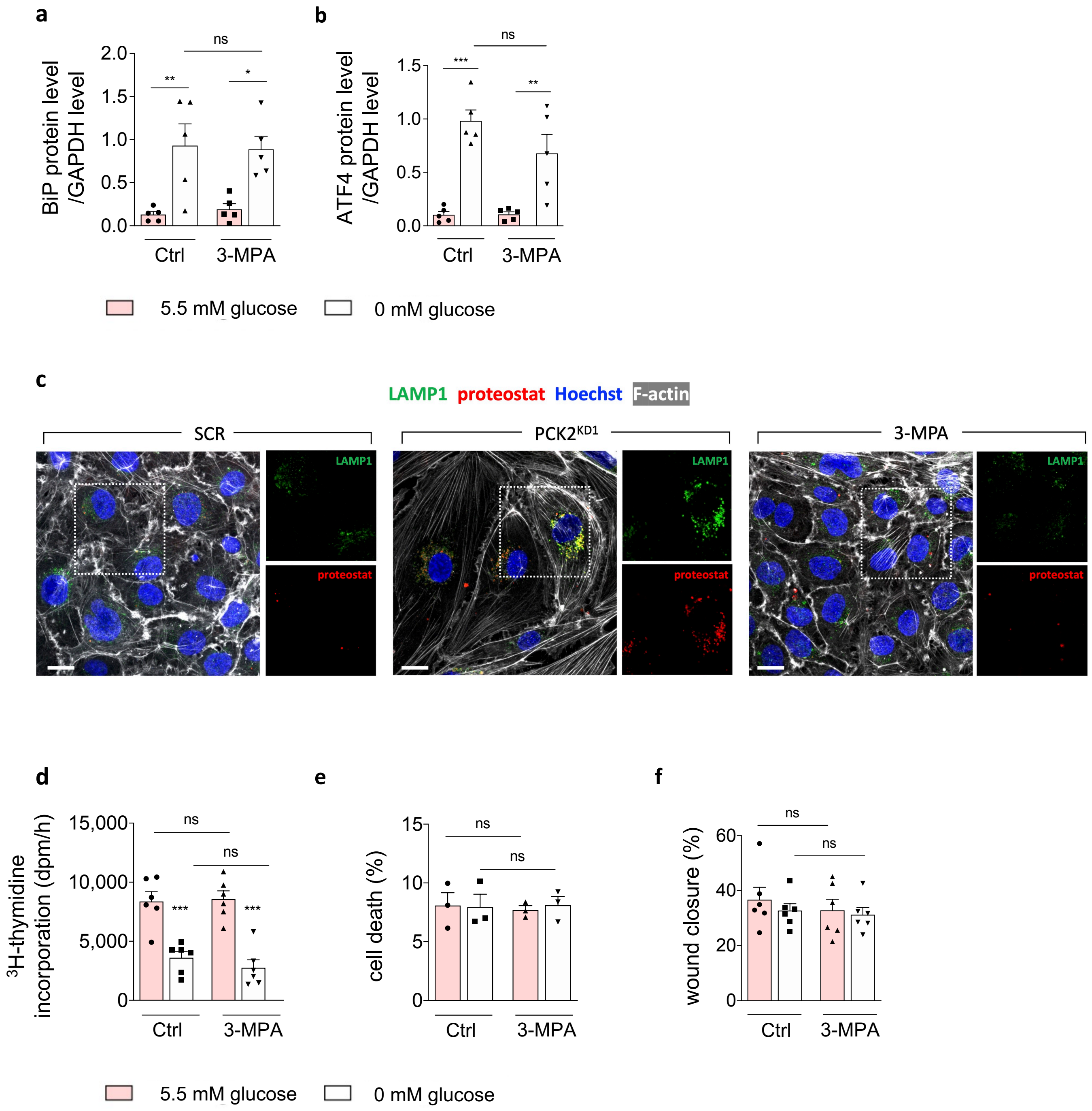
a
BiP protein level /GAPDH level
2.0
1.5
1.0
0.5
0.0
ns
**
*
Ctrl
3-MPA
b
ATF4 protein level /GAPDH level
1.5
1.0
0.5
0.0
ns
***
**
Ctrl
3-MPA
5.5 mM glucose
0 mM glucose
c
LAMP1 proteostat Hoechst F-actin
SCR
PCK2KD1
3-MPA
LAMP1
proteostat
d
3H-thymidine incorporation (dpm/h)
15,000
10,000
5,000
0
ns
ns
***
***
Ctrl
3-MPA
e
cell death (%)
15
10
5
0
ns
ns
Ctrl
3-MPA
f
wound closure (%)
60
40
20
0
ns
ns
Ctrl
3-MPA
5.5 mM glucose
0 mM glucose

SUPPLEMENTARY FIGURE 9: EFFECT OF 3-MPA-MEDIATED CHEMICAL BLOCKADE OF PCK2'S CATALYTIC ACTIVITY ON KEY PHENOTYPIC PARAMETERS

a-b, Densitometric quantifications (Western blots) of BiP (a) and ATF4 (b) protein levels, normalized to loading control (GAPDH), in control or 3-MPA (100 µM)-treated ECs at the indicated glucose concentrations. Data are mean ± s.e.m.; n=5. Statistics: one-way ANOVA; * P<0.05; ** P<0.01; *** P<0.001; ns, not significant. **c,** Representative immunofluorescence images of LAMP1 (green), proteostat (red), F-actin (white) and Hoechst (blue) staining in control (scramble; SCR), $PCK2^{KD1}$ and 3-MPA-treated (100 µM) ECs in 5.5 mM glucose (n=3). Panels on the right are single-channel zoom-ins of the boxed area in the merged images on the left. Scale bar 20 µm. **d-f,** Effect of 3-MPA treatment (100 µM) on proliferation (d), cell death (e) and wound healing (migration) (f) in ECs at 5.5 or 0 mM glucose. Data are mean ± s.e.m.; n≥3. Statistics: one-way ANOVA; *** P<0.001; ns, not significant.

Figure S10 (part 1/3)

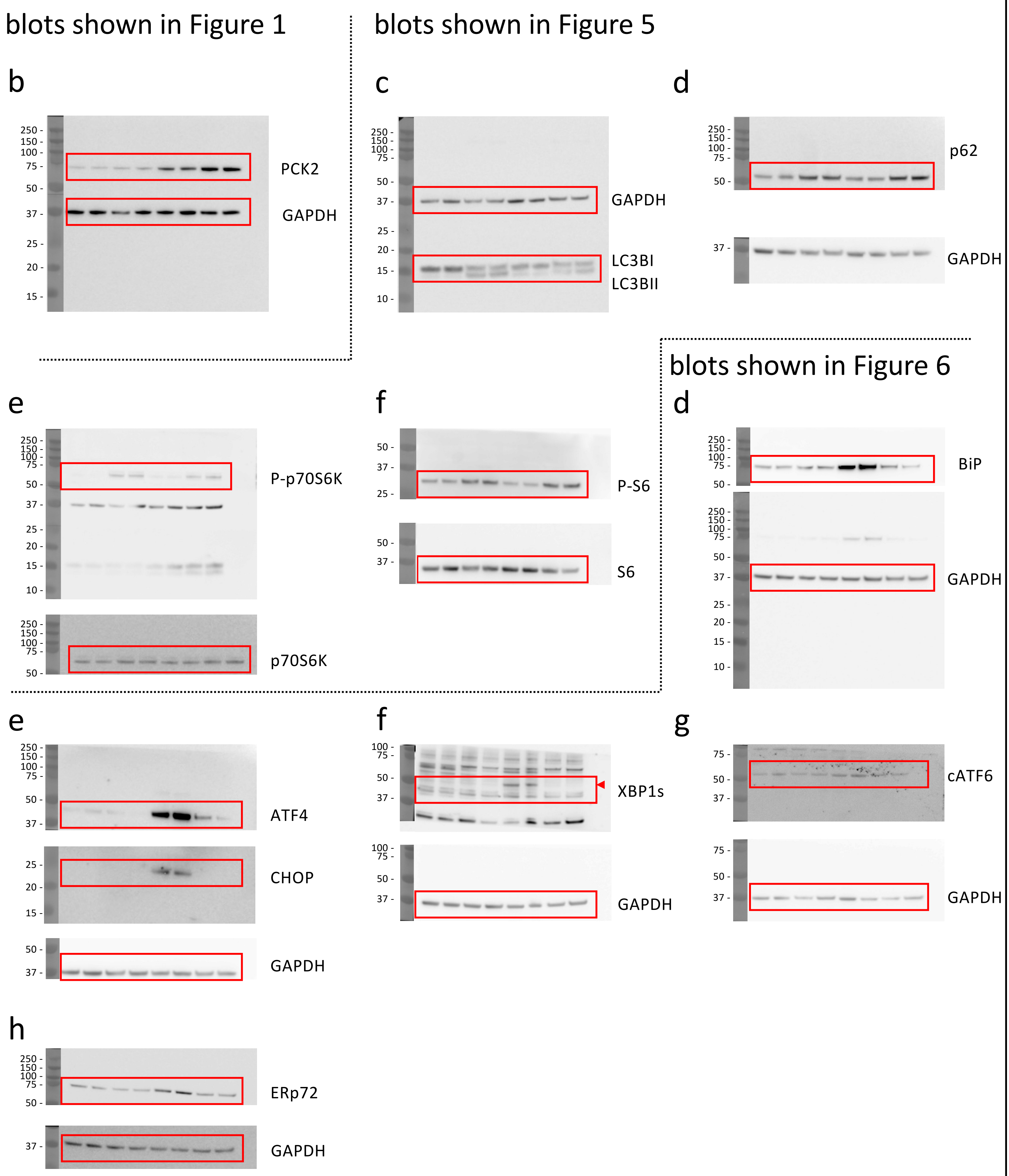

Figure S10 (part 2/3)

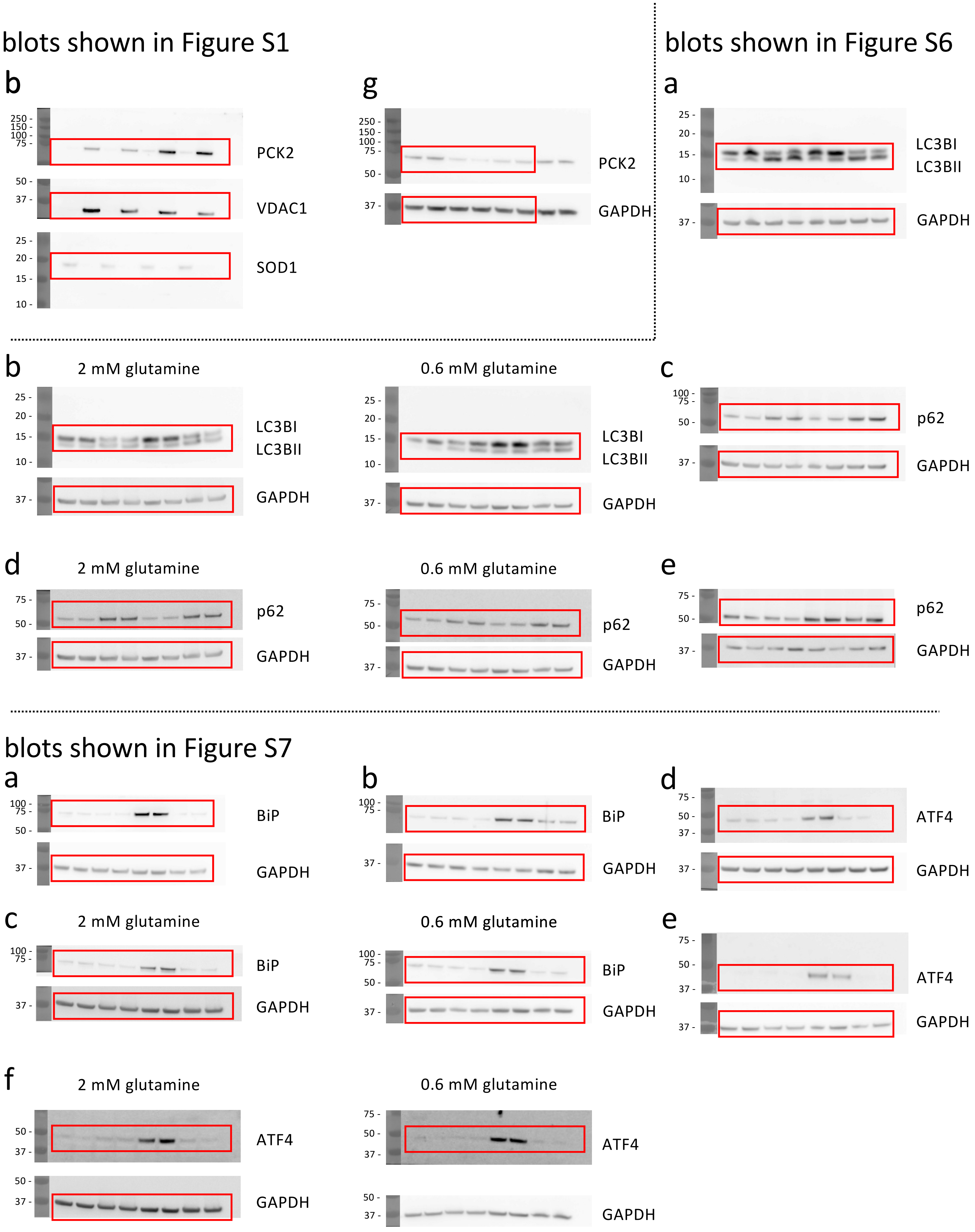

Figure S10 (part 3/3)

blots shown in Figure S8

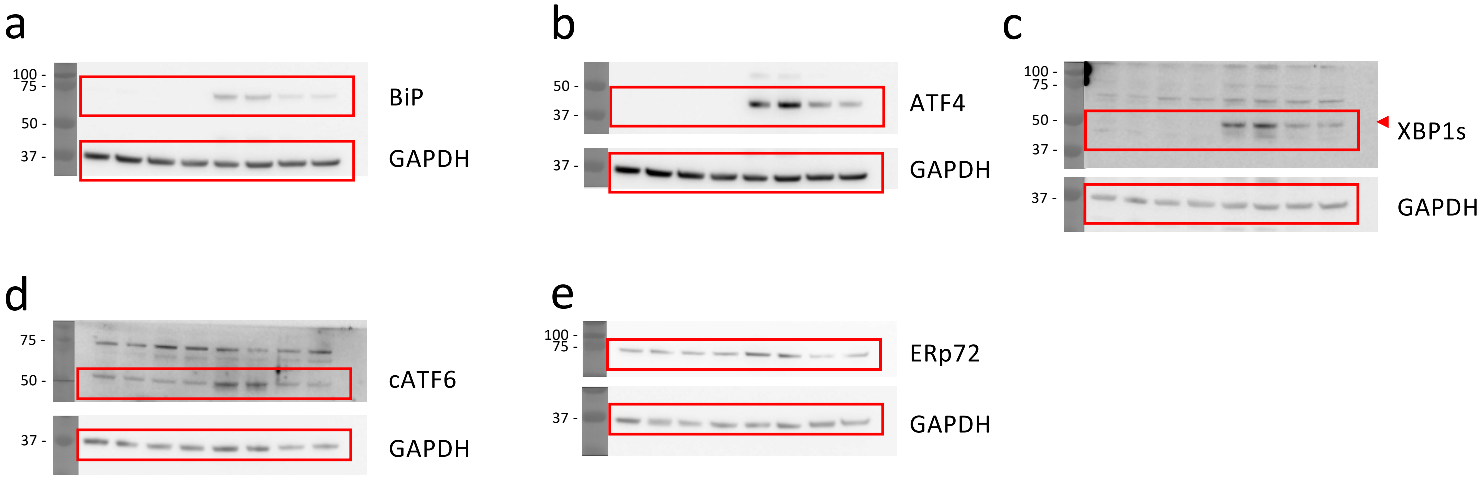

SUPPLEMENTARY FIGURE 10: UNCROPPED WESTERN BLOTS FOR DATA SHOWN IN MAIN AND SUPPLEMENTARY FIGURES.

Individual panels show the uncropped blots with size markers. Red boxes indicate the lanes shown in the figures. Depending on the expected band sizes, blots were cut appropriately to allow for immunoblotting of the respective proteins and loading controls. For space reasons supplementary figures are referred to as 'Figure S'.

SUPPLEMENTARY DATA 1: SOURCE DATA OF THE QUANTIFIED RESULTS OF THE MAIN FIGURES (enclosed excel file)
