# Supplementary material for: The gluconeogenesis enzyme PCK2 has a non-enzymatic role in proteostasis in endothelial cells
Source: Commun Biol. 2024 May 23;7:618. doi: 10.1038/s42003-024-06186-6 (PMC11116505; doi:10.1038/s42003-024-06186-6)
Supplement: Supplementary file 2 — Description of additional supplementary files [file 42003_2024_6186_MOESM2_ESM.pdf]

Description of Additional Supplementary Files

File name: Supplementary Data 1

Description: The source data behind the quantified data in the main figures.
